# Evaluation of the Storage Stability of Starch-Based Films Containing Grape Seed Oil

**DOI:** 10.3390/polym18182279

**Published:** 2026-09-17

**Authors:** Elena-Emilia Sirbu, Bianca Ana-Maria Burdusel, Maria Tănase, Gheorghe Branoiu, Ionut Banu, Catalina Calin

**Affiliations:** 1 Chemistry Department, Petroleum-Gas University of Ploiești, Bucharest Blvd. 39, 100680 Ploiești, Romania; elena.oprescu@upg-ploiesti.ro; 2 National Institute for Research & Development in Chemistry and Petrochemistry ICECHIM, 060021 Bucharest, Romania; 3 Mechanical Engineering Department, Petroleum-Gas University of Ploiești, 100680 Ploiesti, Romania; maria.tanase@upg-ploiesti.ro; 4 Petroleum Geology and Reservoir Engineering Department, Petroleum-Gas University of Ploiești, 100680 Ploiesti, Romania; gheorghe.branoiu@upg-ploiesti.ro; 5 Department of Chemical and Biochemical Engineering, National University of Science and Technology POLITEHNICA Bucharest, 1-7 Gh. Polizu, 011061 Bucharest, Romania; ionut.banu@upb.ro

**Keywords:** potato starch films, grape seed oil, biodegradable packaging, mechanical properties, thermal behavior, crystallinity, sustainable materials

## Abstract

The increasing environmental concerns associated with petroleum-based packaging materials have accelerated the development of biodegradable alternatives, with starch-based films emerging as promising candidates. This study investigates the influence of grape seed oil (5–15 wt.%) and storage time (up to 5 months) on the mechanical, thermal, structural, and surface properties of potato-starch-based films for biodegradable packaging applications. Oil incorporation improved the initial film performance, increasing tensile strength (1.27–3.11 MPa), enhancing thermal stability (melting temperature up to ~118 °C), and promoting higher surface hydrophobicity (contact angle up to ~87°). The differential scanning calorimetry (DSC) analysis indicated time-dependent changes in the thermal transitions associated with the reorganization of ordered starch domains with oil addition, from 39.96% for samples with 5% oil to 36.40% for samples with 15% oil. After three months, all oil-containing samples exhibited a substantial reduction in ultimate tensile strength, with normalized stress values decreasing to 0.74–1.18 depending on oil content. However, after 5 months, the evaluated properties had values close to or above the reference. Statistical evaluation confirmed that storage time is the dominant factor governing property evolution, exceeding the influence of oil concentration.

## 1. Introduction

The widespread dependence on fossil-based polymers for packaging applications, particularly within the food industry, has engendered a profound global environmental and resource challenge [1]. Plastic packaging constitutes a significant fraction of total plastic production, accounting for approximately 26% [2]. While conventional petrochemical plastics offer distinct advantages—including low cost, robust tensile properties, and excellent barrier characteristics against oxygen, carbon dioxide, and water vapor—their extensive usage and subsequent persistence in the environment for centuries pose serious ecological threats to both terrestrial and aquatic systems [1]. This pervasive environmental pollution, coupled with the finite nature and inevitable depletion of global petroleum reserves, necessitates a paradigm shift toward sustainable alternative materials [3]. The transition to “green materials” and the adoption of circular economy principles are no longer merely aspirational goals but are mandated by ecological necessity and increasing consumer demand [4]. Food packaging plastics, in particular, contribute disproportionately to environmental plastic waste, amplifying the urgency for materials that decompose effectively after their intended use [3]. To reduce dependence on limited raw materials and prevent problems in food supply chains, a comprehensive approach to sustainability must prioritize sourcing materials from non-food agricultural waste and include biodegradability for end-of-life processing [4].

Biodegradable polymers, or bioplastics, have emerged as the most compelling substitutes for traditional plastics, especially for short-term applications where packaging material is frequently disposed of alongside food products [1]. By definition, according to the American Society for Testing and Materials (ASTM), these materials undergo degradation through the action of naturally occurring microorganisms, resulting in benign end products such as water, carbon dioxide, inorganic compounds, or new biomass. This mechanism inherently prevents long-term waste accumulation [5].

The native structure of starch is characterized by a dense hydrogen-bonded network, which provides starch-based films with excellent oxygen barrier properties under dry conditions. However, the abundance of hydrophilic hydroxyl (-OH) groups results in high moisture sensitivity and water vapor permeability, leading to poor moisture barrier performance [6,7]. These unique physical and barrier properties have established starch as an essential material in the pursuit of environmentally and human-friendly packaging solutions.

Despite its distinct environmental advantages, native starch suffers from critical functional deficiencies that preclude its use as a standalone packaging material. The primary challenges are related to its mechanical fragility and extreme sensitivity to moisture [8]. Starch films are inherently brittle, exhibiting low elongation at break and a tendency toward retrogradation (recrystallization) over time, particularly in dry conditions, which limits their industrial processability and flexibility [9]. Furthermore, the same highly hydrophilic nature of starch—due to its numerous hydroxyl groups—that facilitates rapid degradation by enhancing water uptake and microbial accessibility [10] simultaneously compromises its function as a protective barrier. Starch films exhibit high water solubility and significant water vapor permeability [6]. This fundamental conflict mandates that materials engineering strategies, such as plasticization and compounding, must be employed to eliminate these functional weaknesses. The goal of these modifications is to ensure adequate mechanical and barrier performance necessary for food preservation without entirely sacrificing the polymer’s high biodegradability.

Plasticizers are critical components in the formulation of starch-based materials, as they are necessary to overcome the polymer’s intrinsic brittleness and improve its thermal processability [9]. Plasticizers operate by increasing the free volume within the polymer matrix and promoting mobility [11]. Glycerol is the most widely adopted plasticizer due to its favorable compatibility with starch, low cost, and proven efficacy in significantly enhancing film flexibility and reducing brittleness [5].

Glycerol exerts its plasticizing effect by disrupting the strong intermolecular hydrogen bonding that exists between adjacent starch polymer chains, substituting these with weaker starch–glycerol hydrogen bonds [12]. This structural relaxation effectively increases chain mobility, which is evident in the substantial increase in the film’s elongation at break. However, this essential mechanical improvement comes at the cost of diminished structural integrity. Studies consistently demonstrate that increasing the concentration of glycerol leads to a sharp decrease in both the tensile strength and the modulus of elasticity of the film, indicating a critical compromise in load-bearing capacity [12].

Although glycerol effectively improves the flexibility of starch films, its hydrophilic nature increases water uptake and moisture sensitivity. As a result, films containing high glycerol concentrations may exhibit increased hygroscopicity and poorer water vapor barrier performance. To address these limitations, research has increasingly focused on incorporating hydrophobic additives, such as essential oils, to improve barrier properties and overall film stability [13]. Grapeseed oil, rich in lipophilic compounds and natural phenolic antioxidants, is proposed as a synergistic component intended to stabilize the starch matrix while conferring active properties to the packaging system [14].

The incorporation of hydrophobic oils offers a pathway to increase the overall material stability and improve the retention of volatile flavor and aroma compounds in packaged foods [12]. Critically, the oil introduces components that can counteract the high surface polarity and increased water affinity caused by the glycerol plasticizer [12]. The introduction of grapeseed oil and its derivatives, rich in phenolic compounds, has been shown to significantly enhance film functionality. The optimized films resulting from such formulations exhibit low water solubility and remarkably reduced oxygen and water permeability coefficients [15]. Grapeseed oil serves a dual-action role: structurally, it may act as a filler or a secondary plasticizer that enhances hydrophobicity, effectively stabilizing the mechanical structure and reducing water diffusion [15]; chemically, its components are effective active agents. Essential oils, including grapeseed extracts, possess a broad spectrum of antimicrobial activity against bacteria, fungi, and yeast, making them potent additives for preventing food contamination and microbial spoilage. For instance, utilizing grape juice-based edible films significantly reduced lipid oxidation (indicated by a decrease in TBARS from 1.98 to 0.91 mg MDA kg^−1^) and achieved measurable reductions in total aerobic mesophilic, psychrophilic, and Enterobacteriaceae counts [15].

Grape seed oil (GSO) has attracted increasing attention as a sustainable additive for biodegradable food-packaging materials due to its renewable origin and valuable chemical composition. As a by-product of the wine industry, GSO contributes to the valorization of agro-industrial residues and supports circular-economy strategies aimed at reducing waste generation and promoting sustainable material development [16]. The oil is rich in polyunsaturated fatty acids, particularly linoleic acid, as well as tocopherols, phytosterols, and phenolic compounds, which are responsible for its antioxidant properties and potential food-preservation benefits [17]. These characteristics make GSO an attractive ingredient for active packaging systems, where the packaging material is expected not only to provide physical protection but also to contribute to maintaining food quality during storage [18]. Furthermore, hydrophobic additives such as vegetable oils have been widely investigated to improve the moisture resistance of starch-based films, which are inherently sensitive to water due to their hydrophilic nature [19]. Recent studies have demonstrated the feasibility of incorporating grape seed oil into biodegradable packaging formulations, where it can provide antioxidant functionality while maintaining the sustainability advantages of bio-based materials [20]. Therefore, grape seed oil represents a promising multifunctional additive for starch-based biodegradable films, combining hydrophobicity, antioxidant activity, and the valorization of winery by-products.

Although starch-based films containing natural oils have been widely investigated for improving flexibility and functional properties, limited information is available on their long-term storage stability and thermo-oxidative behavior [21]. Studies evaluating the evolution of thermal, structural, mechanical, and oxidation resistance during prolonged storage using complementary characterization techniques are few [22,23,24]. Therefore, a comprehensive understanding of the influence of grape seed oil on the long-term stability of starch-based films is still needed. Therefore, the objective of this study was to evaluate the storage stability of starch-based films containing grape seed oil by investigating the evolution of their physicochemical, thermal, thermo-oxidative, structural, surface, and mechanical properties over a five-month storage period. A multi-technique approach, including DSC, TGA, oxidation onset temperature (OOT), FTIR, XRD, contact angle, and mechanical analyses, was employed to provide a comprehensive understanding of the stabilizing effect of grape seed oil during storage.

## 2. Identifying the Research Gap and Hypotheses

**Figure 1:**
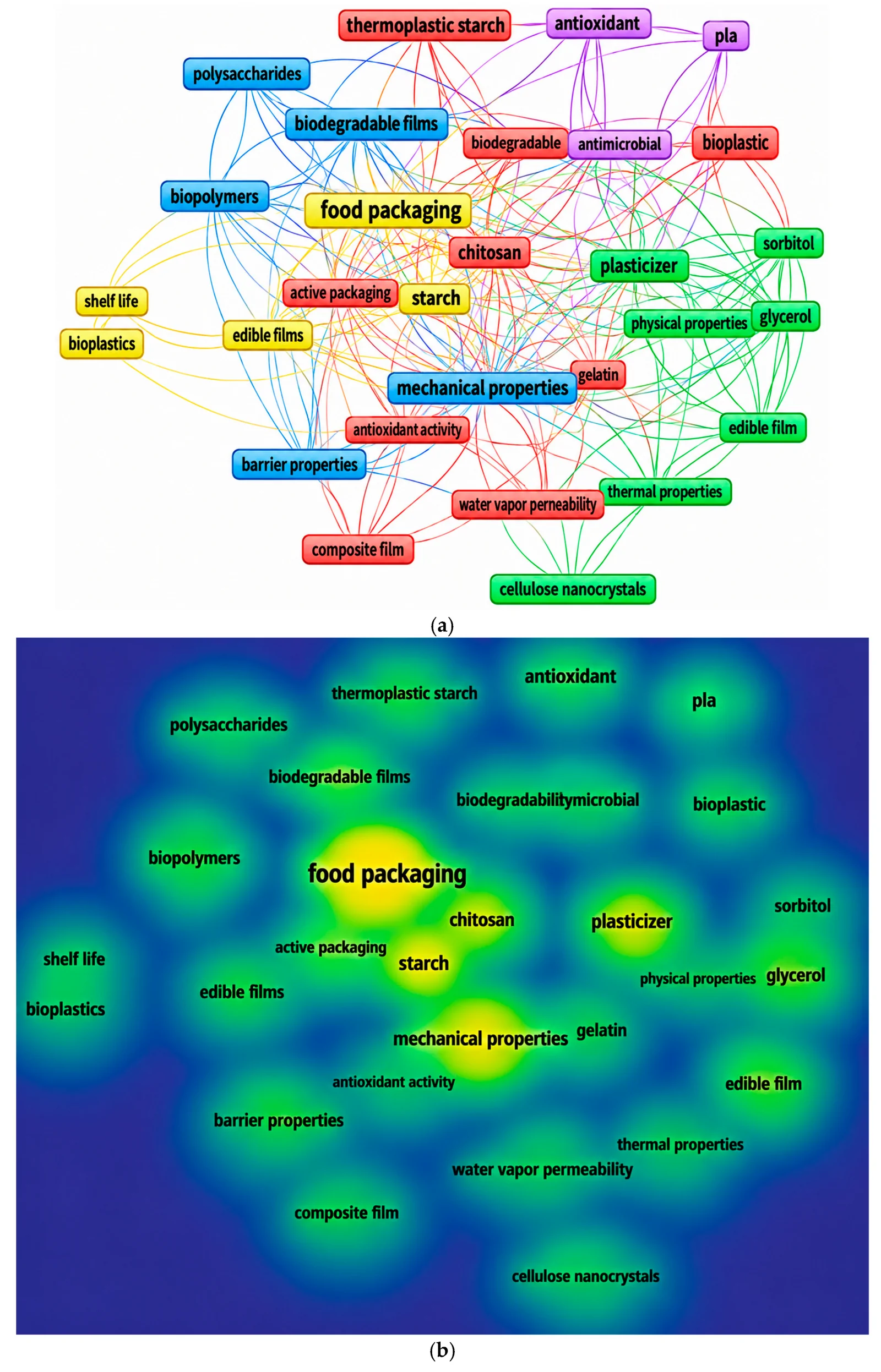

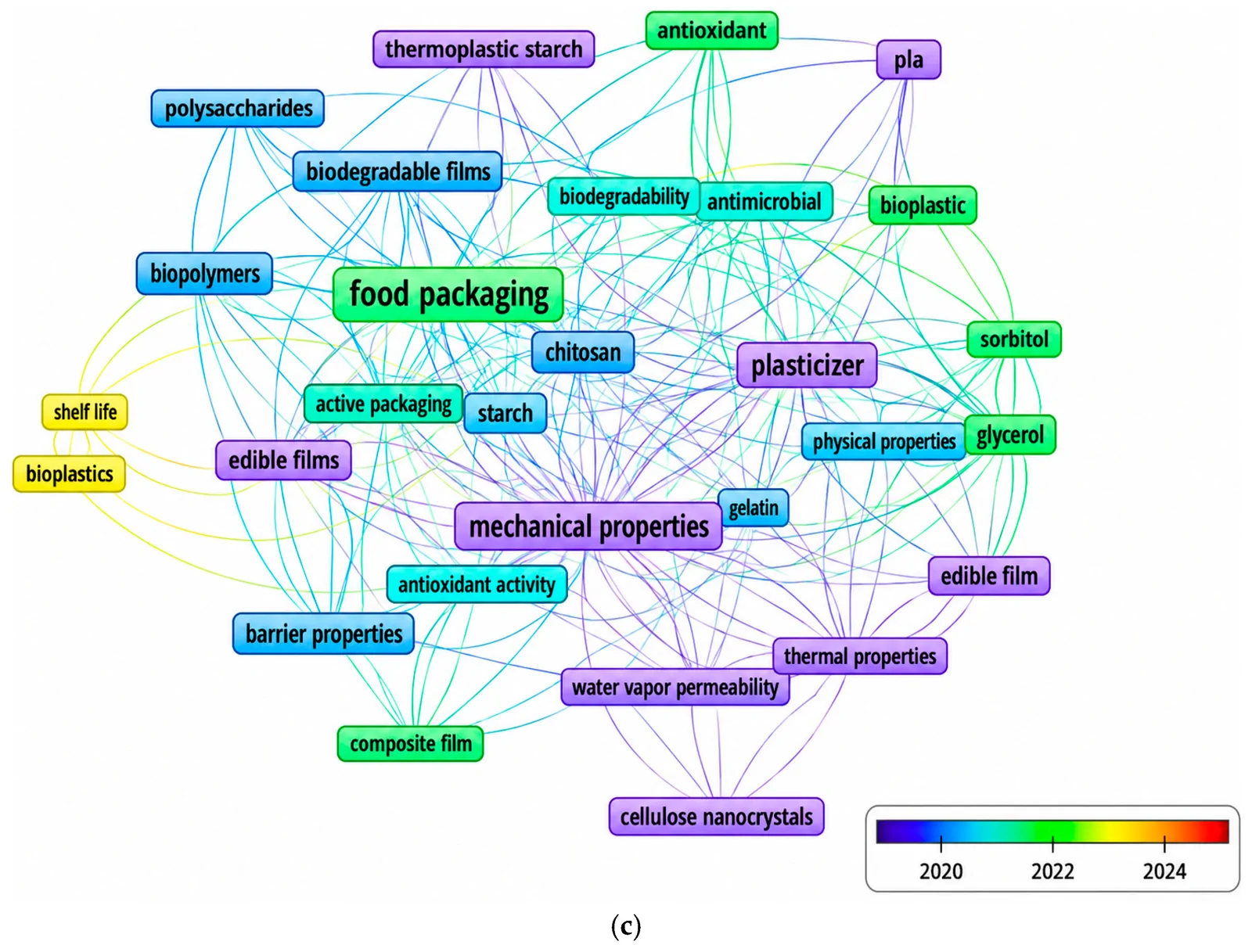
Bibliometric visualizations: (**a**) network visualization; (**b**) density map; (**c**) overlay visualization.

To identify current research trends and gaps, an extensive bibliometric analysis was conducted using the Web of Science (WoS) database. The literature search was performed in December 2025 and included publications between 2015 and 2025. The search query applied was as follows: (TS = (biodegradable films) OR TS = (starch films)) AND (TS = (kinetic) OR TS = (properties)) AND (TS = (plasticizer) AND TS = (Food packaging)). In the search strategy, “TS” refers to the Web of Science “Topic” field. A total of 634 relevant English-language documents were retrieved, including 566 research articles, 2 conference papers, and 3 review articles. A keyword co-occurrence analysis was performed using VOSviewer version 1.6.20, as illustrated in Figure 1, which presents the co-occurrence network map of 40 keywords with a minimum occurrence threshold of 10.

The bibliometric visualizations provide a comprehensive overview of the current research landscape surrounding biodegradable polymers for food packaging. The network visualization (Figure 1a) illustrates the co-occurrence of keywords in the field of biodegradable polymers for food packaging, where each color represents a distinct research cluster. These clusters are formed based on how frequently certain terms appear together in the published literature, reflecting thematic relationships within the field. The yellow cluster, with food packaging at its center, represents the core research focus and acts as the main connecting hub between other clusters. Surrounding this central node are keywords such as starch and edible films, reflecting the dominant research theme of developing environmentally friendly packaging materials based on biodegradable polymers. This cluster emphasizes the growing interest in replacing synthetic plastics with sustainable alternatives suitable for direct contact with food.

The green cluster on the right side of the map focuses on plasticizers and associated keywords like glycerol, sorbitol, physical, and thermal properties. The dense connections between plasticizer, glycerol, and thermal properties indicate that this area has been widely explored, particularly in relation to enhancing film ductility and reducing brittleness.

The blue cluster groups terms such as biopolymers, barrier properties, mechanical properties, and polysaccharides. This cluster bridges materials science and functional performance, reflecting studies that evaluate how polymer composition and additives influence the strength, permeability, and antioxidant characteristics of films. The presence of barrier properties shows a growing but still secondary focus on developing films with improved preservation and protective capabilities for food products.

The red cluster, containing chitosan, biodegradability, antimicrobial, and thermoplastic starch, represents the intersection between natural polymer development and bioactive or antimicrobial functionality. This group of studies often explores the use of polysaccharides and natural biopolymers to enhance biodegradability and inhibit microbial growth. Yet, compared to the central yellow cluster, its connections are thinner, suggesting that antimicrobial and bioactive enhancements are less integrated into mainstream packaging research.

The purple cluster, containing keywords like PLA, antioxidant, and antimicrobial, represents a relatively smaller and more specialized research niche focusing on polylactic acid and related materials. These studies are somewhat peripheral but indicate an interest in alternative biodegradable matrices beyond starch and chitosan.

The density visualization (Figure 1b) highlights where research efforts have been most concentrated, with bright yellow areas marking frequent and central topics, and cooler green or blue tones indicating less attention. The most intense regions appear around food packaging, starch, plasticizer, and mechanical properties, showing that these are the main pillars of this research field.

The strong brightness of starch reflects its key role as the dominant biodegradable polymer, valued for being renewable and cost-effective. Similarly, the high intensity around the plasticizer—particularly glycerol and sorbitol—points to ongoing interest in improving flexibility and thermal performance. The glow near mechanical properties shows that optimizing strength and elongation remains a consistent focus across studies.

Meanwhile, moderately bright zones like physical, thermal and barrier properties suggest steady but less dominant exploration. The cooler blue regions—covering topics such as antioxidant activity, cellulose nanocrystals, and biodegradability—indicate emerging research areas that are gaining attention but remain less developed.

The overlay visualization (Figure 1c) offers insight into how the field has evolved over time. Early studies (dark blue, ≈2020) concentrated on fundamental aspects such as mechanical properties, thermal properties, and water vapor permeability, mainly in starch, chitosan, or gelatin films with plasticizers like glycerol and sorbitol. Mid-stage research (light blue, ≈2021) shifted toward biodegradable films, composite films, and barrier properties, aiming to enhance performance and stability. The next stage (green, ≈2022–2023) centered on food packaging and active packaging, with composite films incorporating glycerol, sorbitol, and antioxidant additives to improve flexibility and functional performance. The most recent trends (yellow, ≈2024) focus on bioplastics and shelf life, highlighting the transition from lab-scale formulations toward sustainable and application-oriented packaging solutions.

## 3. Materials and Methods

### 3.1. Materials

Potato starch and all chemicals were of analytical grade (Merck, Darmstadt, Germany). The grape seed oil (Costa d’Oro, Spoleto, Italy) was bought from a local store in Ploiesti, Romania.

### 3.2. Starch Film Preparation

The starch films were prepared according to Sirbu et al. [24], with some modifications. Starch films were prepared by adding 5 g of potato starch, 30 mL of distilled water, 4.5 g of a 9 wt.% (*w*/*w*) aqueous acetic acid solution, and 2.5 g of glycerin and grape seed oil (GSO) in concentrations of 5, 10 and 15% relative to the amount of starch. The reaction mixture was homogenized with an IKA T18 ULTRA TURRAX (IKA, Staufen, Germany) at a rotation speed of 20,000 rpm for 30 min, after which it was heated to approximately 100 °C (boiling temperature) under continuous stirring. Finally, the solution mixtures were poured into circular glass Petri dishes (90 mm in diameter) and labeled according to Table 1. The films were kept at room temperature for 3 and 5 months before characterization and testing.

### 3.3. Characterization of Starch Films

The methods used for determination of solubility, swelling degree, contact angle and opacity were described in a previous study of our research team [24] (Supplementary Materials). For each test, three repetitions were performed. The thermal properties of the film samples were assessed using the thermogravimetric/derivative apparatus TGA/DTG (TGA 2 Star System Mettler Toledo, Zurich, Switzerland) in the temperature range 25–600 °C at a rate of 10 °C per minute in a controlled nitrogen atmosphere. Differential scanning calorimetry (DSC) determination was performed with a DSC 3+ Star system from METTLER TOLEDO (Leicester, UK), under a N_2_ atmosphere at 10 °C/min between 25 °C and 250 °C. The influence of grape seed oil addition on the oxidative stability of films was evaluated using the oxidation onset temperature (OOT) measured via differential scanning calorimetry (DSC) [25]. Meanwhile, the crystallization behavior of the materials was analyzed by XRD analysis described in study [26]. The mechanical properties of the samples were tested using Lloyd Instruments equipment with a maximum load capacity of 2.5 tons. Tensile tests were conducted on the specimens at a grip displacement rate of 20 mm/min. The methods used for the determination of film swelling degree, film solubility in water, water contact angle, and opacity are described in detail in the Supplementary Materials.

It should be noted that the control starch film (SF-0-0) was characterized only at the initial stage of the study. During storage, the control film rapidly underwent starch retrogradation, resulting in a significant loss of flexibility and structural integrity after approximately one month. Consequently, the films became too brittle to be handled and tested reliably under the same experimental conditions used for the grape seed oil-containing formulations.

## 4. Results and Discussion

### 4.1. The Opacity

The opacity of a film can be influenced by variations in composition, density, and the aggregation or alignment structure of biopolymer molecules, which alter the refractive index and limit light transmission through the film matrix. Thus, higher opacity is associated with reduced spacing between film components, leading to a more compact structure. Lipid (oil) addition decreases transparency and increases opacity. Films with oil laminated between layers are more opaque compared to those without oil [27].

**Figure 2:**
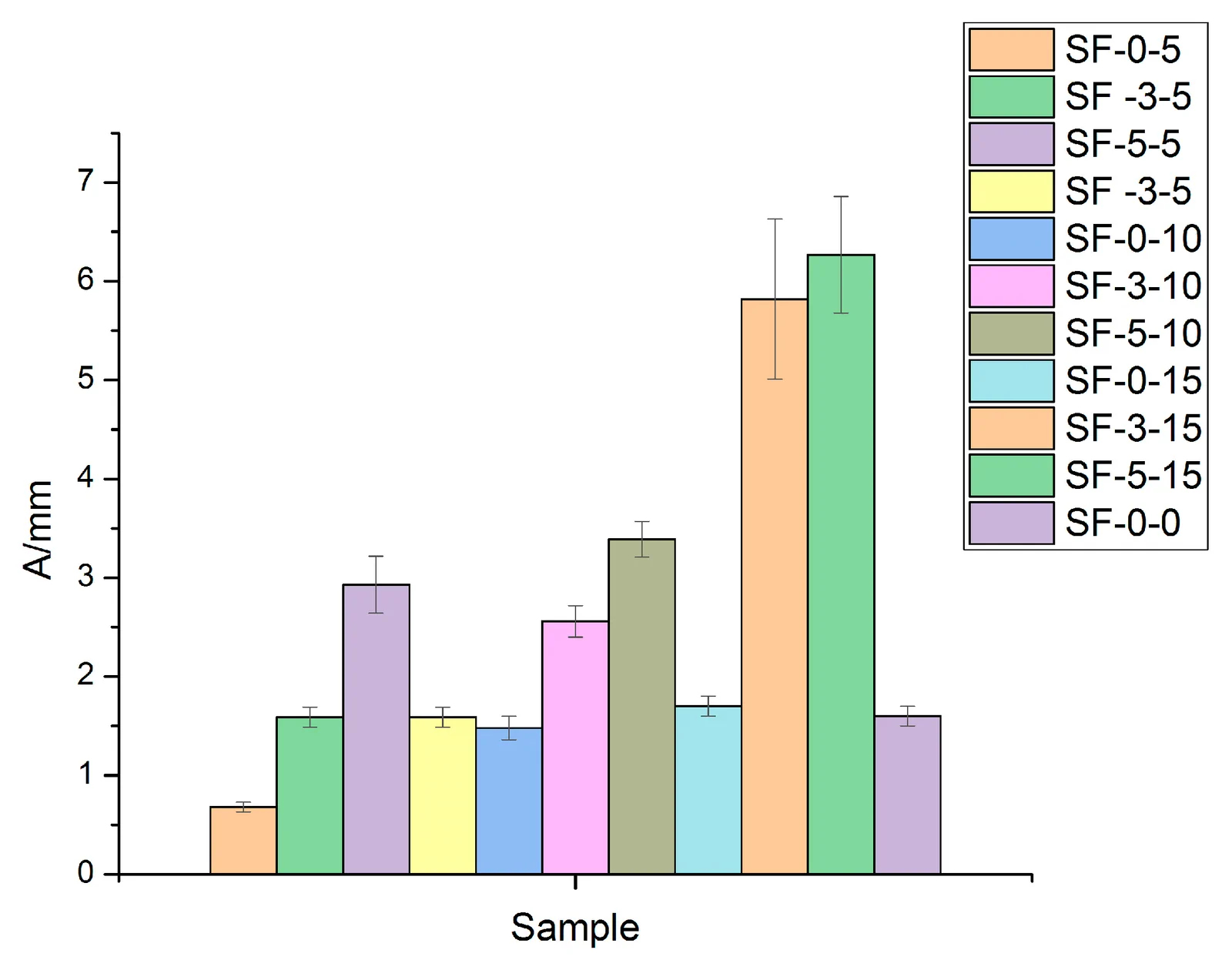
The opacity data of starch films.

In Figure 2, an increase in opacity can be observed as the oil concentration in the composition increases, rising from 0.68 in the sample containing 5% GSO (SF-0-5) to 1.7 for the sample with 15% GSO (SF-0-15), which is consistent with data reported in the literature [28,29].

According to Justyna Kadzińska et al. [30], the addition of vegetable oils results in a significant increase in opacity values compared to the control film, which also correlates with visual observations. Xiaoyong Song and collaborators showed that the incorporation of lemon essential oil decreases transparency and increases both the haze and opacity of the films as the oil concentration increases [30]. Regarding the storage of biopolymer films containing GSO, the opacity level increased significantly after 3 months for all samples: the film containing 5% GSO (SF-3-5), 10% GSO (SF-3-10), and 15% GSO (SF-3-15). Among the films stored for 3 months, increasing the GSO concentration resulted in the largest increase in opacity for the sample containing 15% GSO, which reached an opacity value of 5.82. This increase in opacity over time in starch-based films can be attributed to moisture migration and internal structural changes, as well as phase separation processes occurring in multicomponent starch–oil systems. These transformations enhance light scattering through the film, thereby reducing its initial transparency [31]. After a storage period of 5 months, a slight increase in opacity can be observed for all three studied samples, SF-5-5, SF-5-10, and SF-5-15, reaching a maximum value of 6.27 for the sample containing 15% GSO (SF-5-15). In addition, a lower opacity value was observed for the sample without oil (SF-0-0) after 5 months of storage compared to the sample containing 5% oil (SF-5-5). This behavior can be explained by the fact that during storage, starch-based films undergo retrogradation processes that lead to the reorganization of amylose and amylopectin chains. As the storage time increases, the polymer network becomes progressively more ordered and eventually approaches a relatively stable structure, resulting in slower changes in optical properties such as opacity [32,33].

### 4.2. The Solubility

The solubility of the polymers depends on the chemical composition, average molecular weight, chain flexibility, temperature, crystal structure and degree of crystallinity of the polymer. The higher the molecular weight, the lower the solubility of polymers in the same solvent [24].

**Figure 3:**
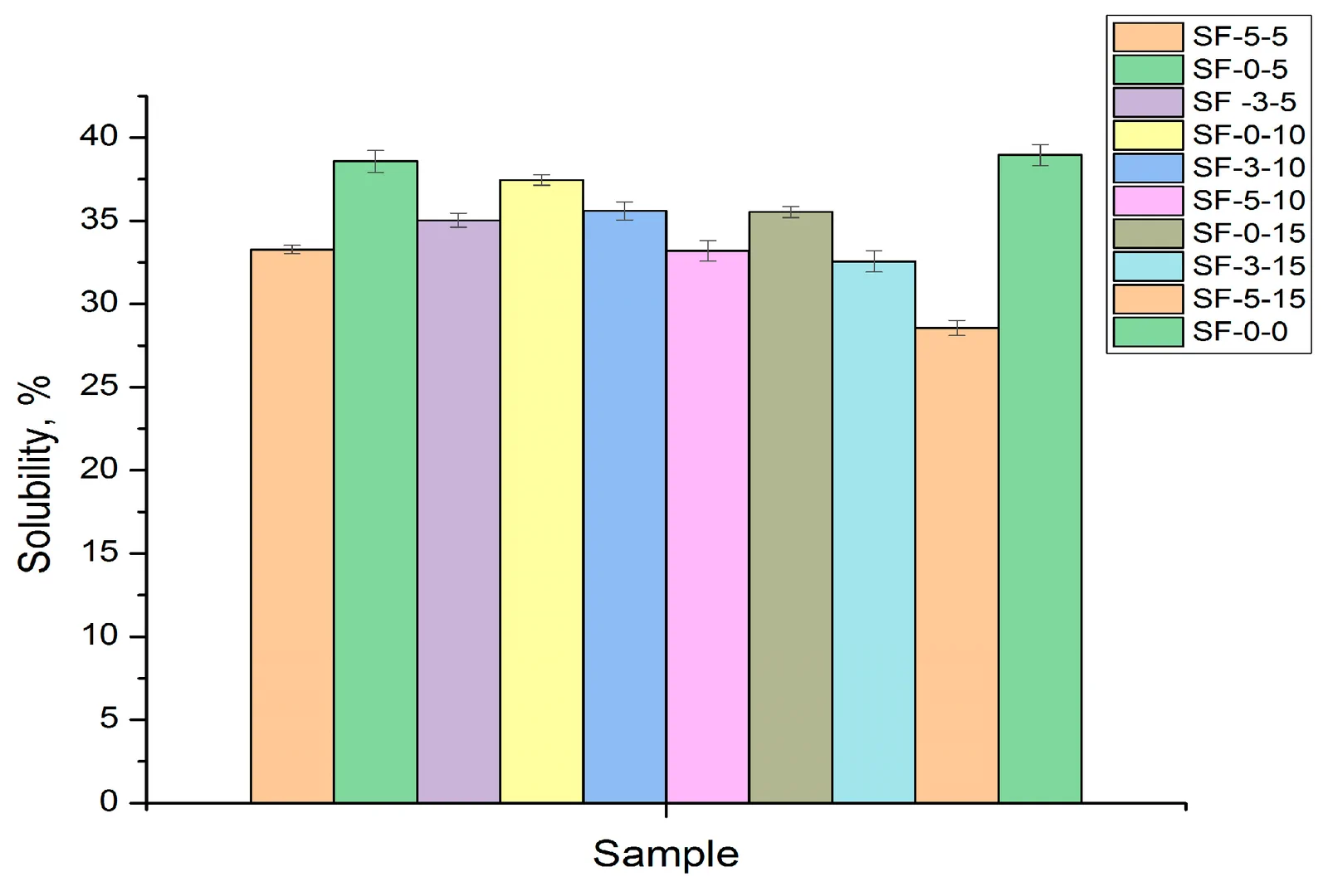
The influence of GPO on solubility of starch films.

As shown by the data presented in Figure 3, the presence of glycerin and oils in biopolymer-based films influences their solubility. When oil (grape seed oil, GSO) is incorporated into starch-based films, water-related properties and solubility can be affected [34,35]. In the case of fresh samples, where grape seed oil was added at proportions of 5%, 10%, and 15%, a slight decrease in solubility was observed with increasing oil content, from 38.57% to 35.52%. This suggests that the oil acts as a hydrophobic agent, reducing the ability of the biopolymer to dissolve in water. According to Mutlu [36], the addition of oil introduces porosity and hydrophobic components. Furthermore, the incorporation of oils or hydrophobic compounds into starch/gluten-based films reduces their solubility in water by decreasing the interaction between hydroxyl groups and water molecules, and increasing the hydrophobicity of the polymer matrix [37]. With regard to variations in solubility after 5 months from the preparation of biopolymer films, a decrease in solubility was observed for the sample containing 5% oil, from 38.57% (SF-0-5) to 33.27% (SF-5-5), which is consistent with findings reported in the literature [24]. Similarly, the solubility of biofilms containing 10% oil decreases over time from 37.45% (SF-0-10) to 33.19% (SF-5-10). The same trend is observed for samples with an oil content of 15%, where solubility varies from 35.52% (SF-0-15) to 28.56% (SF-5-15) after 5 months from preparation. As the oil content in the sample increases to 15% (SF-0-15), solubility continues to decrease moderately after 5 months (SF-5-15), as reported by Fatma Nur Akgül et al. [38]. This behavior is explained by the formation of starch–oil complexes (including those with hydrolyzed forms of oil), which are less soluble than native starch under certain conditions.

The decrease in solubility of starch- and oil-based polymer films over time is a commonly observed phenomenon, caused by various physicochemical changes that occur as the material undergoes aging [39]. Starch retrogradation (or recrystallization) refers to the process in which amylose and amylopectin chains (the main components of starch) reassociate and reorganize into a more crystalline structure after gelatinization [40]. As a result, the structure becomes more ordered, more hydrophobic, and less permeable to water, which leads to a reduction in solubility [9].

In conclusion, the solubility of starch-based films with a low oil concentration (grape seed oil, GSO 5%) decreases slowly over time because the starch is well dispersed and undergoes gradual degradation [41]. As reported by Li et al., when a moderate concentration of added oil (10%) is used, solubility decreases more rapidly because the oil begins to interact with the starch chains, forming microaggregates that reduce solubility [42]. As the oil concentration incorporated into the starch biofilm increases to 15%, solubility decreases rapidly, reaching a value of 28.56% (SF-5-15), as a result of the formation of starch–lipid complexes. In addition, emulsion instability further accelerates the decrease in solubility, which is consistent with data reported in the literature [38,43].

### 4.3. Swelling Index

During the swelling process, the molecular structure of starch transitions from a tightly packed, ordered arrangement to a more relaxed and disordered state, ultimately facilitating the dissolution of starch molecules. Water penetrates the amorphous regions of the granules, disrupting intermolecular interactions and increasing the mobility of polymer chains, which contributes to the breakdown of the crystalline structure [44]. Furthermore, water absorption and granule swelling lead to the weakening of hydrogen bonds between double helices, the opening of crystalline domains, and the separation of polymer chains [45].

**Figure 4:**
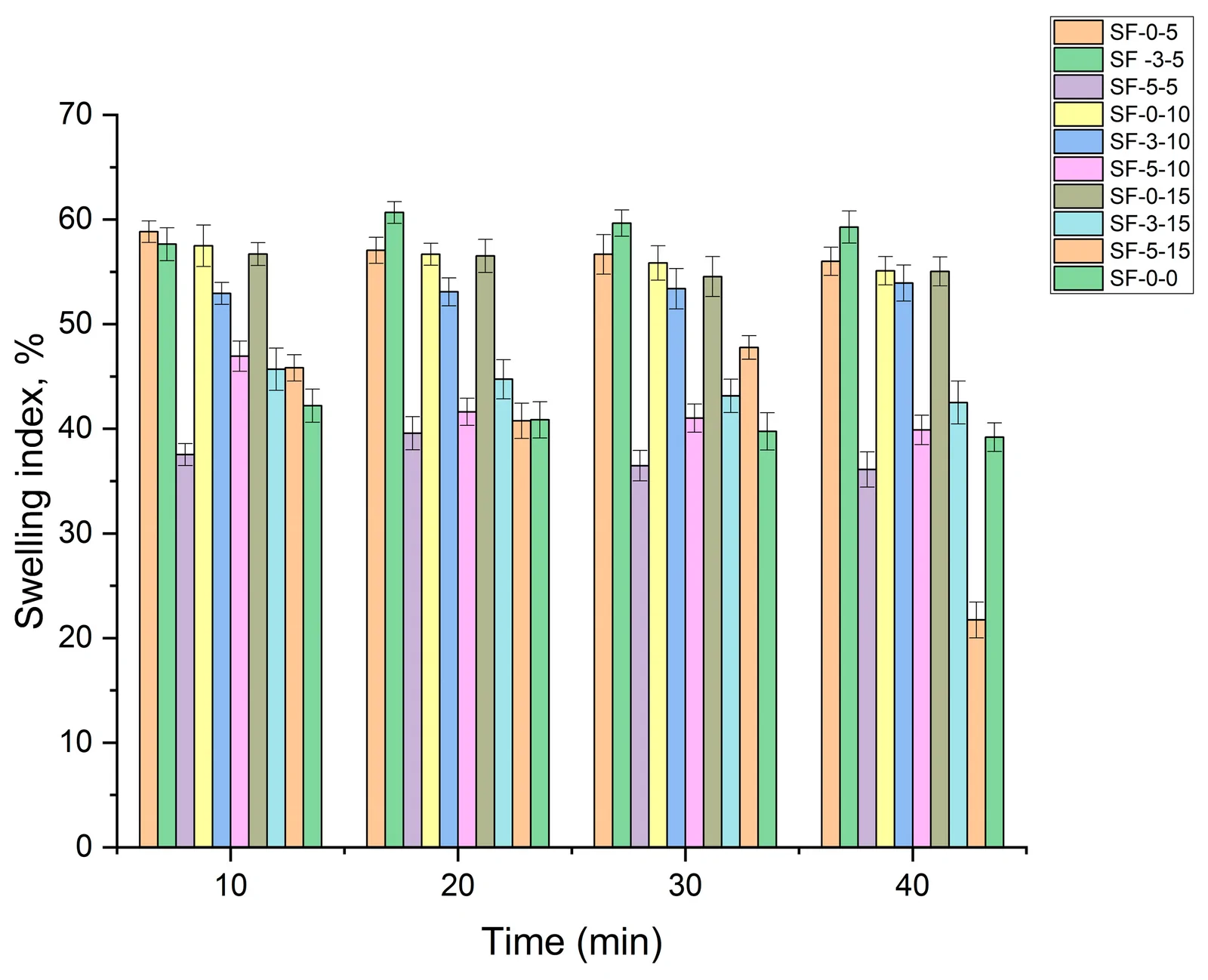
Film swelling index.

As seen in Figure 4, in cases of samples containing oil (5%, 10%, and 15%), the swelling degree decreases with increasing oil concentration, ranging from 58.83% to 56.69% after 10 min, with no significant variations as a function of swelling time. This behavior suggests the existence of a balance between the hydrophilic and hydrophobic segments of the matrix. According to Mehran Dolat Khah et al. [46], the addition of oil reduces the interaction between the film and water, which may also indicate a decrease in the swelling degree. The low solubility reflects a less water-permeable network, which can be associated with a lower swelling degree [47]. Furthermore, Hooman Chodar Moghadas et al. [28] demonstrated that the incorporation of hydrophobic components (such as oil) tends to reduce swelling (i.e., water absorption), provided that the film allows interaction with water.

Regarding the evolution of the swelling degree over time, the following observation can be made: the lowest swelling degree recorded was 21.73% (SF-5-15 after 40 min), measured after 5 months of storage for the film variant with the highest GSO concentration (15%), due to the formation of strong hydrogen bonds within the biopolymeric structure. It can thus be concluded that the swelling degree decreases over time as a result of structural reorganization processes and the formation of additional intermolecular bonds [48], as well as with increasing content of non-hydrophilic components in the biopolymer structure, due to the densification of the polymer network [49].

### 4.4. TGA Analysis

**Figure 5:**
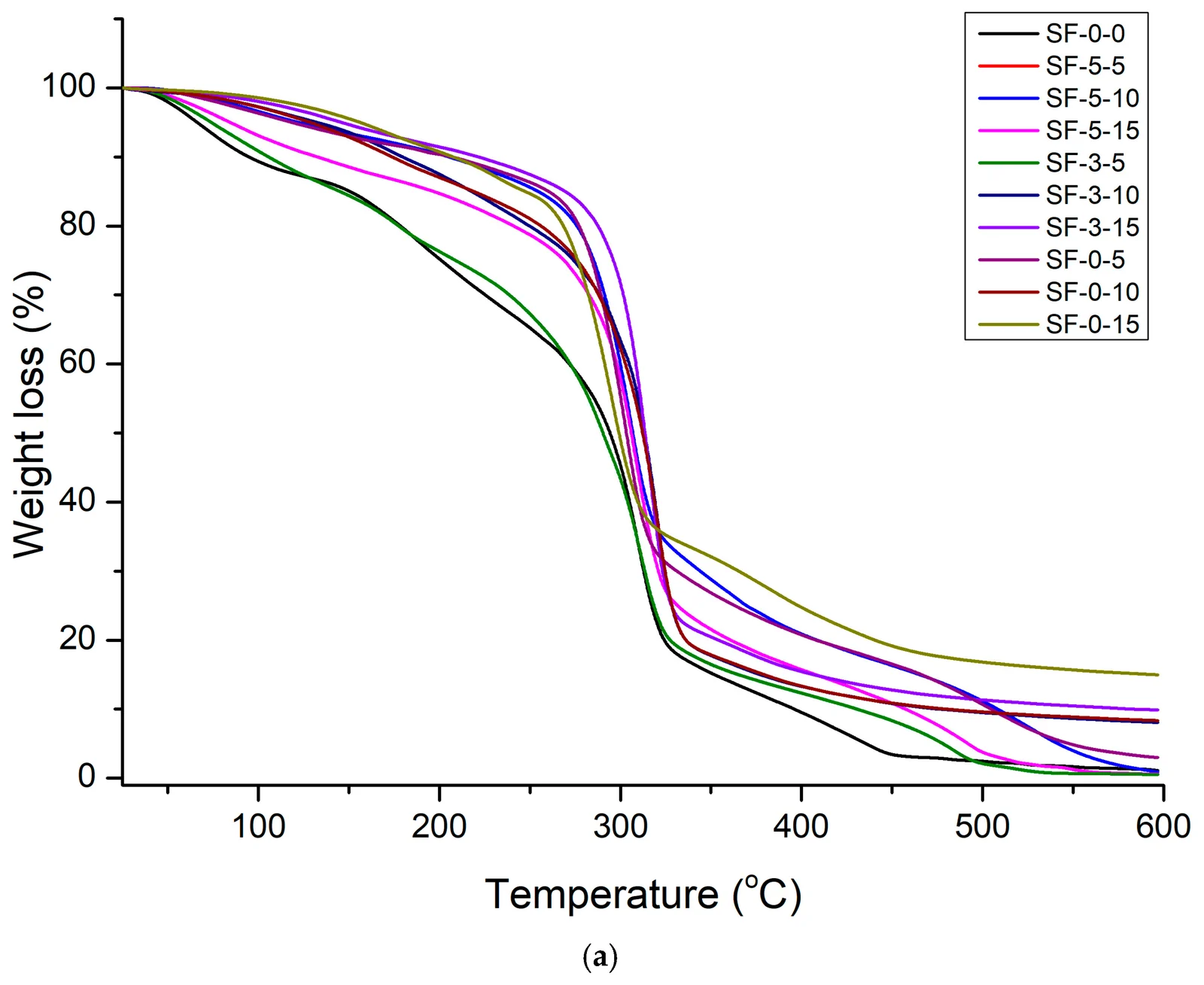

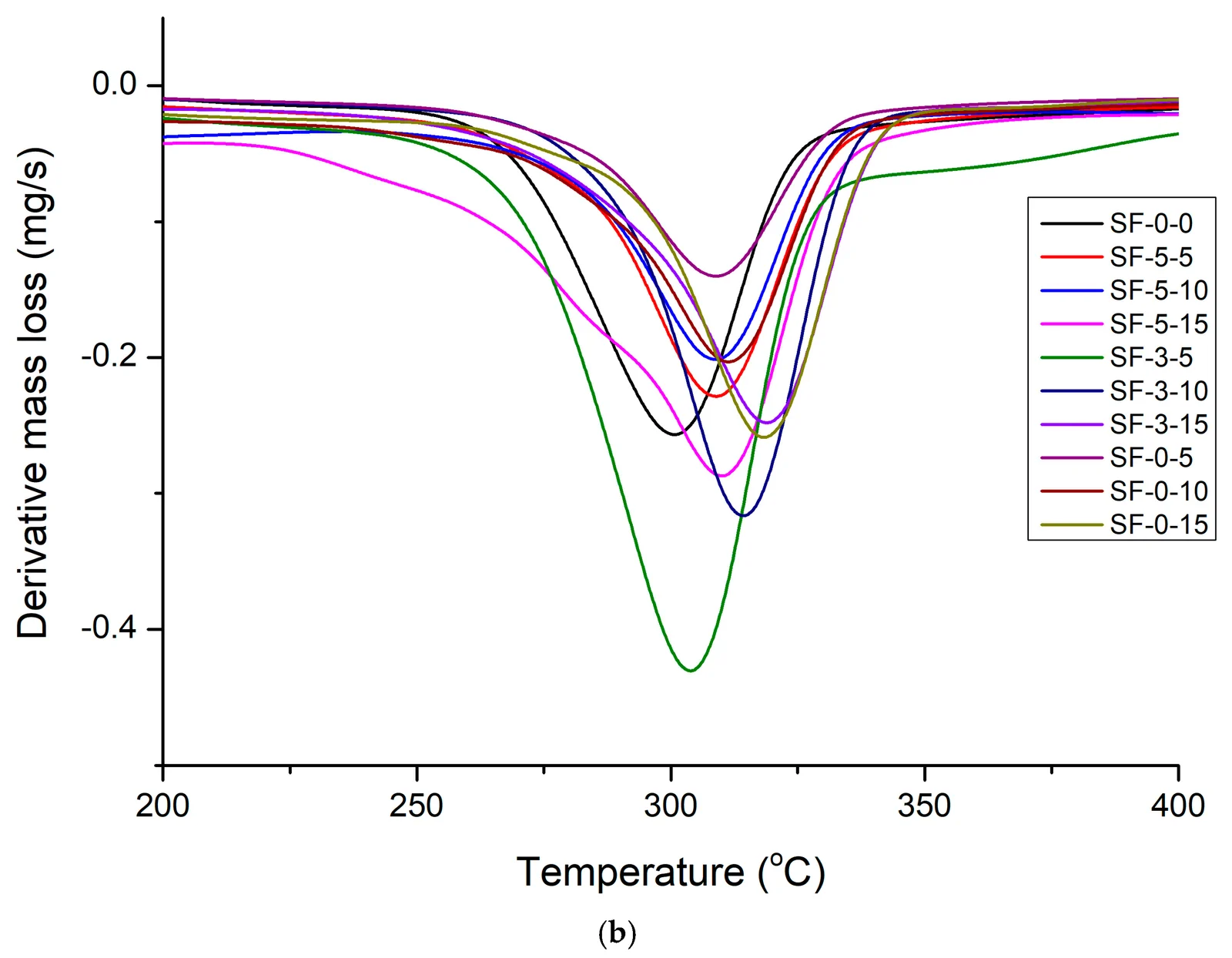
Thermogravimetric analysis of starch film: (**a**) thermogravimetric analysis; (**b**) derivative thermogravimetric analysis.

The TGA and DTG graphs indicate that the thermal degradation of potato starch occurs in two separate phases (Figure 5). Stage I (30–120 °C) shows a moderate mass loss of 1.59% to 10.97%, due to the evaporation of weakly bound adsorbed water inside the starch polymer film. The control sample exhibits greater mass loss; meanwhile, the samples with 15% oil had a mass loss between 1.59 and 2.86%, indicating that the presence of oil influences moisture retention, acting as a hydrophobic barrier. The second stage (120–380 °C) is caused by the thermal degradation of grape seed oil, glycerol and starch polysaccharides, specifically amylose and amylopectin. This phase is characterized by a major weight loss between 66.37 and 79.21%. In the DTG, the degradation peaks can be seen at 294 °C for the SF-0-0 sample and 319 °C for the SF-0-15 sample. The enhanced thermal stability of the composite film can be attributed to several complementary mechanisms [50]. First, the hydrophobic lipid phase reduces the affinity of the starch matrix for water, as evidenced by the lower mass loss observed during the first degradation stage. Reduced moisture content decreases thermally induced plasticization effects and delays thermal degradation. Furthermore, the dispersed oil phase may promote physical interactions between lipid molecules and starch chains, leading to a more compact structure and restricting polymer-chain mobility. Similar increases in thermal stability have been reported for starch-based films containing fatty acids, vegetable oils, and oleoresins, where the incorporation of hydrophobic compounds shifted degradation temperatures toward higher values due to improved matrix cohesion and reduced water sensitivity [27]. In addition, tocopherols and phenolic compounds present in grape seed oil may contribute to thermo-oxidative stability by scavenging free radicals and interrupting oxidative chain-propagation reactions generated during heating in the presence of oxygen [16,51,52,53]. These combined effects explain the increase in T_max_ from 294 °C for the control film to 319.33 °C for the film containing 15% GSO, confirming the stabilizing effect of grape seed oil on the starch matrix [24,54]. The highest residual mass, around 32%, corresponded to the starch film containing the highest concentration of grape seed oil. The results obtained (Table 2) highlight that the addition of grape seed oil significantly influences the thermal behavior of potato starch films. By increasing the maximum degradation temperature, the oil acts as a thermal stabilizer, improving the thermal resistance of the films, indicating a relatively high resistance to heat exposure [55]. Regarding the evolution of thermal stability over time, it is observed that it decreases, regardless of the added oil concentration, but remains above the value of the SF-0-0 sample. For example, for samples containing 5% oil, T_max_ decreases from 309.17 °C after 3 months to 301 °C after 5 months. This decrease may be caused by the chemical and physical degradation of starch or migration of plasticizers (glycerol and grape seed oil) over time under the action of environmental factors such as humidity, temperature or light [28]. This statement can be supported by the residual mass that decreases over time; for example, for samples containing 15% oil, the residual mass decreases from 32.04% to 21.05 after 5 months.

### 4.5. DSC Analysis

**Figure 6:**
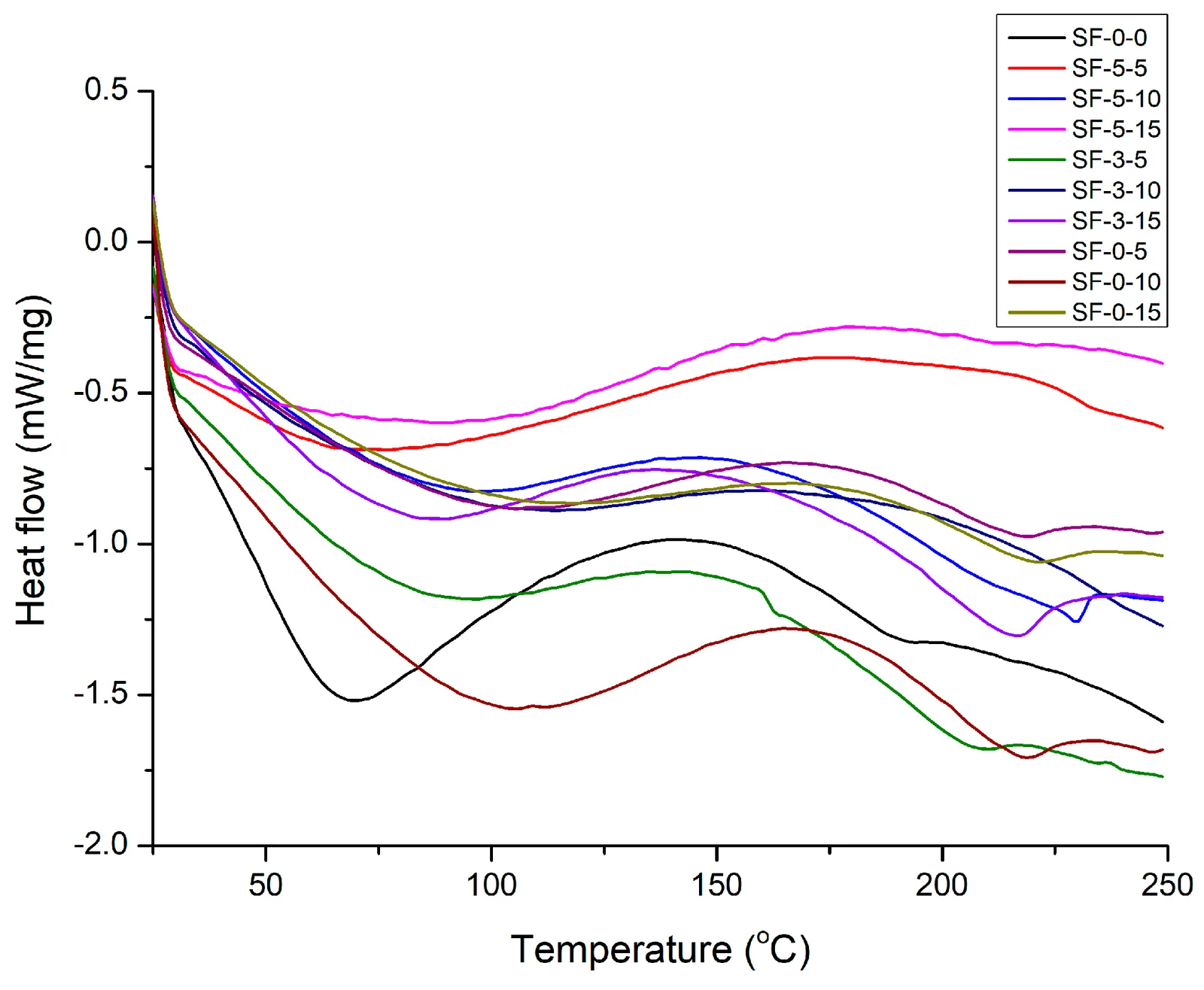
DSC thermograms of starch films.

DSC analysis data for the films, as illustrated in Figure 6, indicate that all samples exhibit a noticeable broad endothermic peak within the temperature range of approximately 65 °C to 190 °C. This peak is attributed to gelatinization of starch during the film production process [56]. The endothermic event observed between 210 and 230 °C is attributed to structural rearrangements and disruption of ordered starch domains rather than to the main thermal degradation process. The actual decomposition of the films occurs at higher temperatures, as evidenced by the TGA/DTG results (T_max_ = 294–319 °C) [57]. The samples showed a higher melting temperature (T_m_) as the concentration of grape seed oil increased. For instance, the T_m_ value of SF-0-5, SF-0-10 and SF-3-15 films, which contain 5, 10 and 15% oil, increased from 104.5 °C to 117.63 °C and to 118.49 °C, respectively. Enhanced thermal stability of the crystalline regions indicated that the internal movement of the molecular chain was limited due to the interaction between amylose–amylopectin–lipid chains, which led to the formation of a stable starch–lipid structure that required high temperatures for melting [58]. Also, the heterogeneous nature of starch–lipid complexes was indicated by the broader gelatinization temperature range observed for all the samples containing grape seed oil [59,60]. As observed and explained in TGA analysis, upon storage, starch retrogradation and plasticizer migration take place in all samples, leading to a decrease in T_m_ temperature and an enhancement of crystallization degree. For example, for samples with 5% oil, T_m_ decreased from 104.5 °C for fresh samples to 84.72 °C after 3 months and finally to 78.27 °C after 5 months (however, it remains higher than samples without grape seed oil (68.57 °C)); meanwhile, the crystallization degree increases from 20.68% (SF-0-5) to 24.89% (SF-3-5) and 39.96%.

The influence of grape seed oil addition on the oxidative stability of films was evaluated using the oxidation onset temperature (OOT) measured via differential scanning calorimetry (DSC) [25]. The increase in oxidation onset temperature (OOT) with increasing grape seed oil content indicates that the lipid phase delays the initiation of thermo-oxidative degradation. This behavior can be attributed to two complementary mechanisms. First, grape seed oil contains natural phenolic compounds and tocopherols that act as antioxidants, thereby delaying lipid oxidation and increasing the oxidation onset temperature [61,62]. Second, interactions between starch macromolecules and phenolic compounds, together with starch–lipid associations, reduce polymer chain mobility and hinder oxygen diffusion through the polymer matrix, resulting in enhanced thermo-oxidative stability [63]. These mechanisms are consistent with the experimental results obtained in the present study, where the oxidation onset temperature increased from 60.54 °C for the control film to 68.73 °C, 77.00 °C, and 93.81 °C for films containing 5, 10, and 15 wt.% GSO, respectively. Likewise, the DSC results showed an increase in melting temperature from 68.57 °C (SF-0-0) to 118.49 °C (SF-0-15), indicating the formation of a more thermally stable starch–lipid network. During storage, the OOT gradually decreased (from 93.81 °C to 91.57 °C after 3 months and 87.07 °C after 5 months), which can be attributed to starch retrogradation, plasticizer migration, and progressive oxidation of the unsaturated lipid fraction. Nevertheless, all GSO-containing films maintained higher thermo-oxidative stability than the control sample, demonstrating that grape seed oil provides sustained protection against thermo-oxidative degradation despite the aging process.

### 4.6. Contact Angle

**Figure 7:**
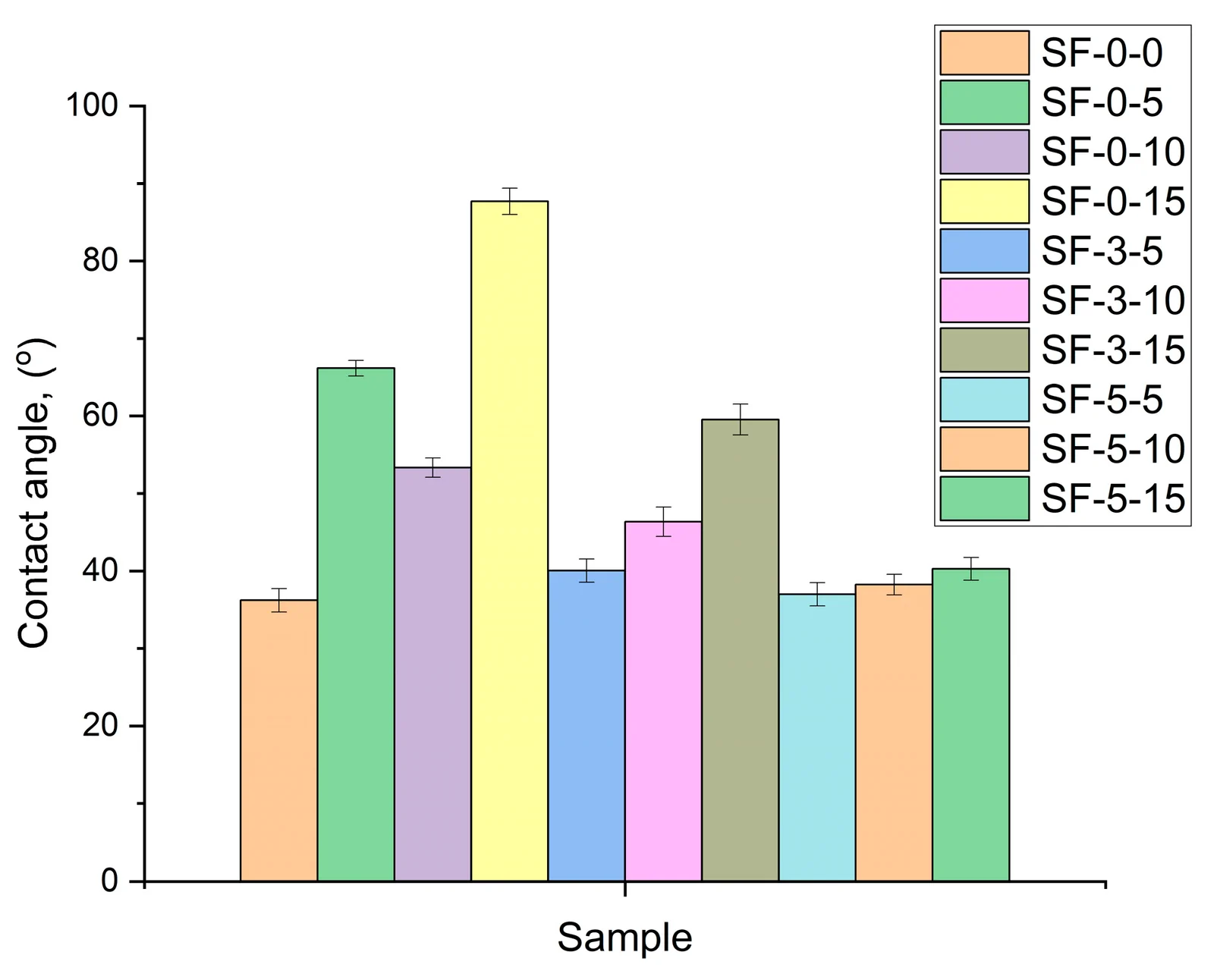
Contact angle data and water drop images of fresh starch films containing different concentrations of GSO.

The contact angle indicates the relationship between the surface tensions of air, liquid, and the film surface. A higher contact angle indicates a greater surface affinity of films for liquids that are less polar than water [64]. As can be seen from Figure 7, for the fresh samples, the addition of grape seed oil increased the contact angle from 66.17° for SF-0-5 to 87.7° for SF-0-15, indicating an increase in surface hydrophobicity [65]. Nonetheless, over time, the contact angle values diminished for all samples (Table 3). For instance, samples with the highest oil content (E3) exhibited a decrease in contact angle to 59.54° and 40.27° after 3 months and 5 months, respectively. This behavior indicates that the lipophilicity of starch films diminished due to alterations in molecular configuration resulting from the breakdown of grape seed oil, leading to a distinct spatial arrangement [66].

### 4.7. XRD Analysis

**Figure 8:**
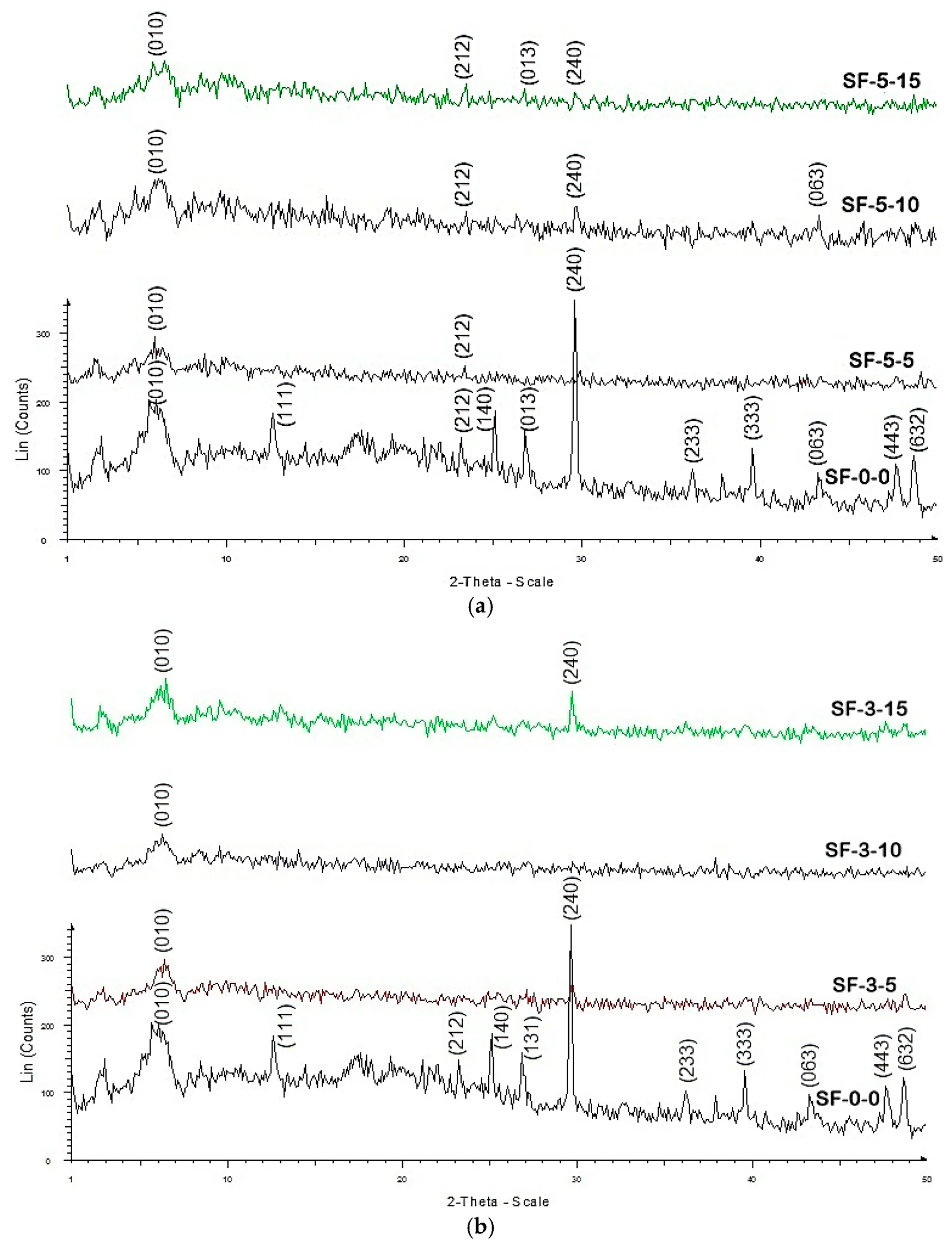

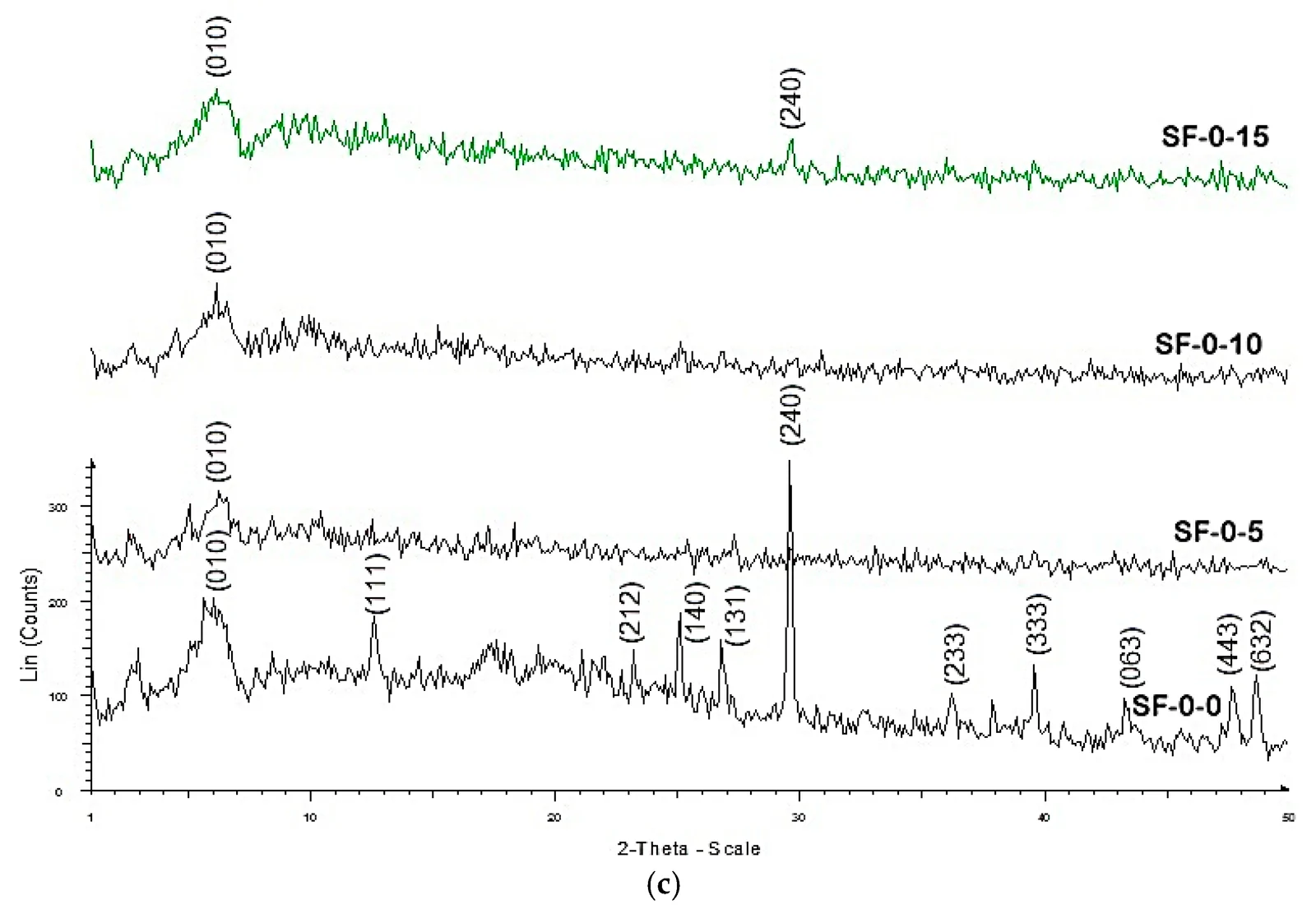
XRD spectra of starch films after different storage times: (**a**) 5 months; (**b**) 3 months; (**c**) 0 months. The SF-0-0 pattern corresponds to the oil-free control measured at the initial time point (t = 0) and is reproduced in panels (**a**,**b**) solely as a reference for comparison with the GSO-containing films after 5 and 3 months of storage, respectively.

X-ray diffraction analysis was used to evaluate the degree of structural order and the changes induced by the presence of plasticizer (GSO) and storage time on potato starch films. The diffractograms obtained for the control sample (SF-0-0) and for the plasticized samples (SF-0-5, SF-0-10, SF-0-15, SF-3-5, SF-3-10, SF-3-15, SF-5-5, SF-5-10, SF-5-15) at times t = 0, t = 3 and t = 5 months are presented in Figure 8. It should be noted that the SF-0-0 diffractogram shown in the 3- and 5-month panels corresponds to the control sample measured at the initial time point (t = 0) and is included solely as a reference to facilitate comparison with the GSO-containing films during storage; no additional XRD measurements of the control sample were performed after 3 or 5 months.

The control sample (SF-0-0), representing the starch film without oil, presents a pronounced semi-crystalline characteristic, with well-defined reflections corresponding to the diffraction planes (010), (111), (240), (333) and (632). The crystallinity value calculated for SF-0-0 is 22.87% (XRD), which is the highest value in the analyzed sample set. This indicates a superior structural organization of the starch chains in the absence of the plasticizer [67,68,69].

The incorporation of GSO into the starch matrix (samples SF-5-5, SF5-10, and SF-5-15) leads to a significant modification of the diffraction spectra. A decrease in the intensity of the reflections and their broadening are observed: a phenomenon accompanied by a drastic reduction in the degree of XRD crystallinity to values between 9.21% and 11.25%. This behavior is characteristic of the plasticization effect, where GSO molecules interpose between the macromolecular starch chains, reducing intermolecular hydrogen bond interactions and favoring the increase in the amorphous fraction [24,69,70,71,72].

The evolution of the structural parameters over time (0–5 months) indicates a progressive reordering of the polymer matrix. From the data presented in Table 4, a general trend of a slight increase in XRD crystallinity is observed for all plasticized samples: for sample E1 (5% GSO), the crystallinity increases from 9.21% to 11.11%; for sample E2 (10% GSO), high stability is observed, with the final value being 11.76%; for sample E3 (15% GSO), the increase is the most pronounced, from 10.07% to 12.48%. This increase in crystallinity over time is attributed to the starch retrogradation process, by which amylose and amylopectin chains tend to slowly reassociate into ordered structures [73,74,75,76].

The retrogradation of starch after gelatinization is explained by the spontaneous association of β-amylose chains in hexagonal double helices [77]. The increased molecular mobility, favored by a higher GSO (E3) content, accelerates this structural reordering process. The crystal lattice parameters (a = b and c) remain relatively constant over the 5 months, suggesting that the basic crystal arrangement type does not undergo major qualitative transformations, but only a quantitative evolution of the degree of ordering.

In conclusion, samples with an intermediate GSO content (10% oil) present the best overall structural stability, while samples with a high plasticizer content (15%) are the most susceptible to molecular reorganization processes and loss of thermal stability over time. Another important observation derived from this study is that structural changes can influence mechanical properties in the sense that increasing XRD crystallinity could lead to a slight increase in the stiffness over time of starch films, in agreement with previous research [78,79,80,81].

### 4.8. FT-IR Analysis

**Figure 9:**
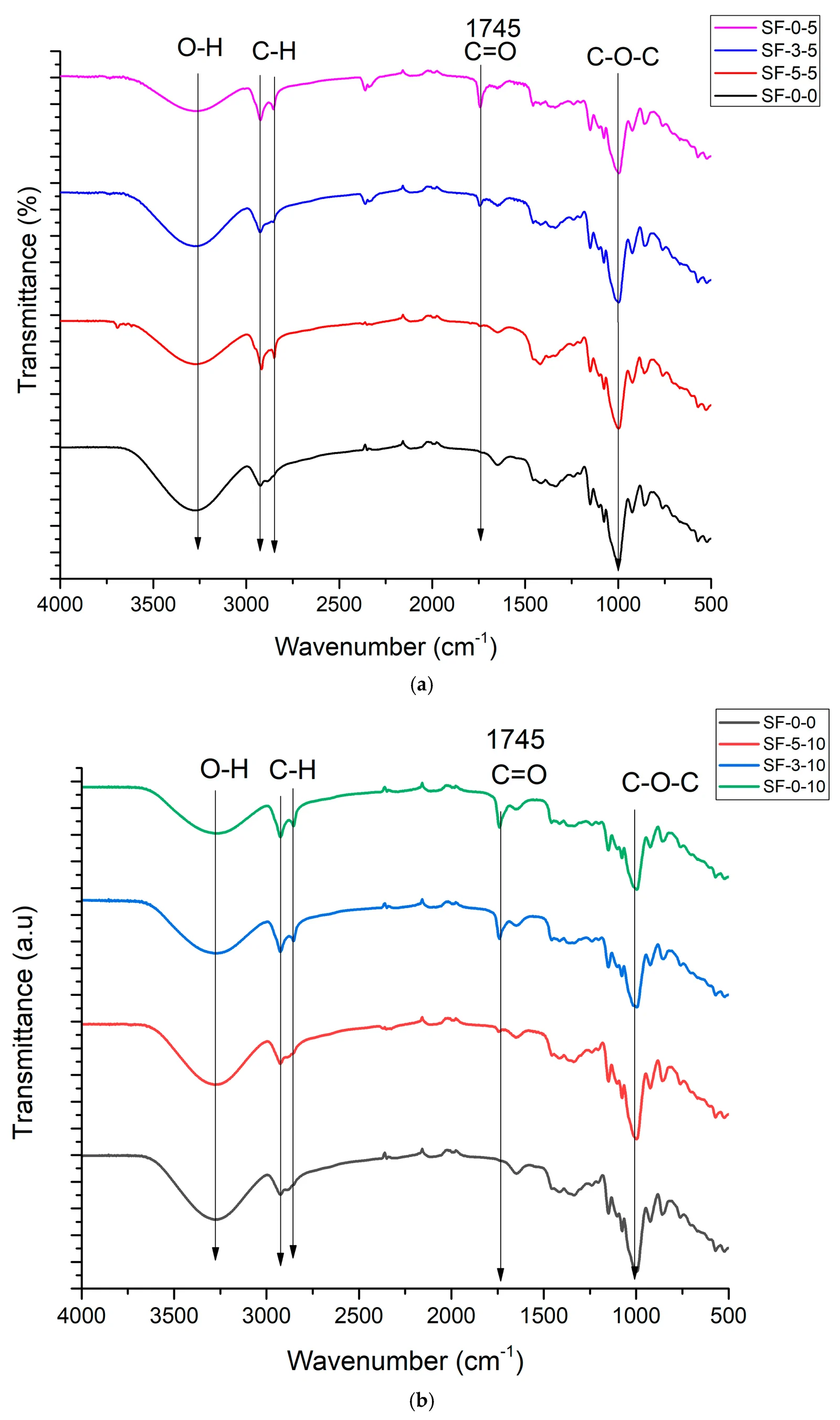

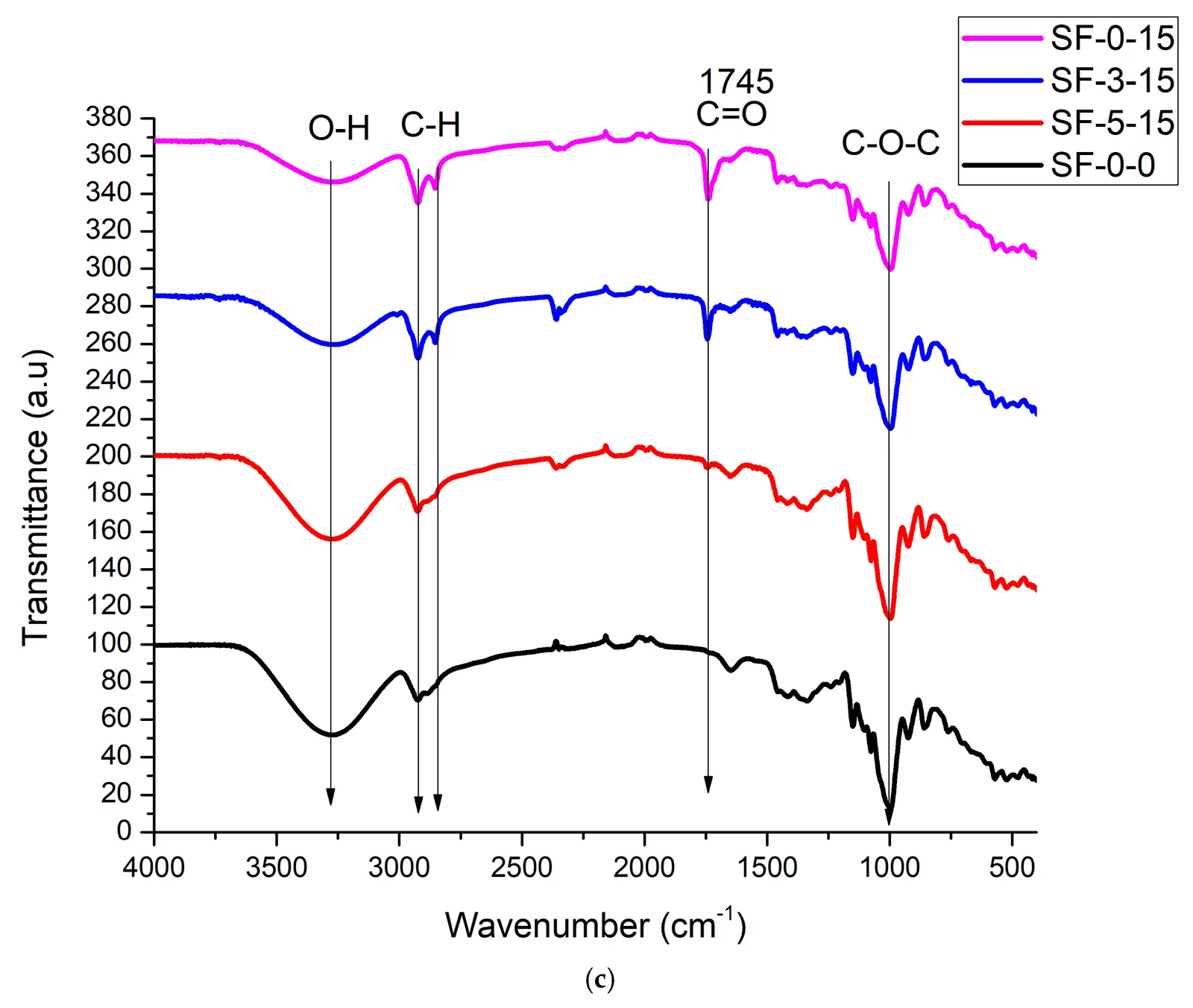
FTIR spectra of the starch films after different storage times: (**a**) 5% oil; (**b**) 10% oil; (**c**) 15% oil.

As can be observed in Figure 9, all samples exhibit absorption bands at 3277 cm^−1^, 2924 cm^−1^, and 1406 cm^−1^, assigned to –OH groups, –C–H stretching in CH_2_, and symmetric deformation vibrations of –CH_3_, respectively. The peak at 1643 cm^−1^ indicates the presence of water. Over time, the FTIR spectra reveal changes associated with structural reorganization processes and the physical aging of starch-based films. A decreasing trend and/or modification of the band corresponding to O–H groups (~3000–3600 cm^−1^) is observed, correlated with the reorganization of the hydrogen-bonding network and the dynamics of water within the system. The band around ~1640 cm^−1^, attributed to water bending vibrations, reflects the migration and release of water from the starch matrix during retrogradation [82]. The region between 1120 and 900 cm^−1^ is typically identified in carbohydrates and polysaccharides, while vibrations in the range of 1147–1078 cm^−1^ are attributed to C–O–H bonds in starch [83]. The spectra exhibited an intense peak at 991 cm^−1^, consistently observed for all films, confirming the polysaccharide nature of the starch-based film matrix [65]. Over time, changes occur in the intensity and ratios between bands (e.g., 1047/1022 cm^−1^ or 1042/1016 cm^−1^), which are directly correlated with an increase in structural order and the formation of double-helix structures during storage [84]. These variations confirm the retrogradation process, characterized by the reassociation of amylose and amylopectin chains and the transition toward a more stable and ordered structure [85]. At the same time, retrogradation is accompanied by the expulsion of water from the polymer network, as evidenced by changes in the ~1640 cm^−1^ band, associated with the disruption of starch–water hydrogen bonds and the formation of new intermolecular starch–starch interactions. This process contributes to increased rigidity and reduced mobility of polymer chains [82]. Compared to the control sample, several bands exhibited different intensities. The intensity of O–H bond vibrations decreases with increasing oil concentration, suggesting interactions between the nonpolar constituents of the oil and the polysaccharide chains, leading to reduced film hydrophilicity. This observation is consistent with contact angle data. The bands at 2922 and 2852 cm^−1^, associated with C–H bond vibrations, become more pronounced and increase in intensity with increasing oil concentration, possibly due to enhanced Van der Waals forces arising from short-range molecular interactions between unsaturated bonds and the polymer matrix [86]. For samples containing a lipid phase, time evolution may also include processes of reorganization or partial phase separation, as well as possible oxidation reactions in unsaturated compounds, reflected by changes in the band characteristics of C–H and C=O groups. Although these effects depend on the specific system, they are frequently reported in starch–lipid composite materials [87]. The sample containing oil shows an additional band at 1745 cm^−1^, characteristic of C=O bonds in the ester groups of triglycerides; compounds absent in the sample are composed exclusively of starch [88]. Therefore, the FTIR spectral analysis suggests that the functional groups within the films interact with each other, contributing to improvements in their stability [89].

### 4.9. The Tensile Testing

Mechanical properties were determined using three replicate specimens for each formulation, and the reported values represent the average of these measurements.

**Figure 10:**
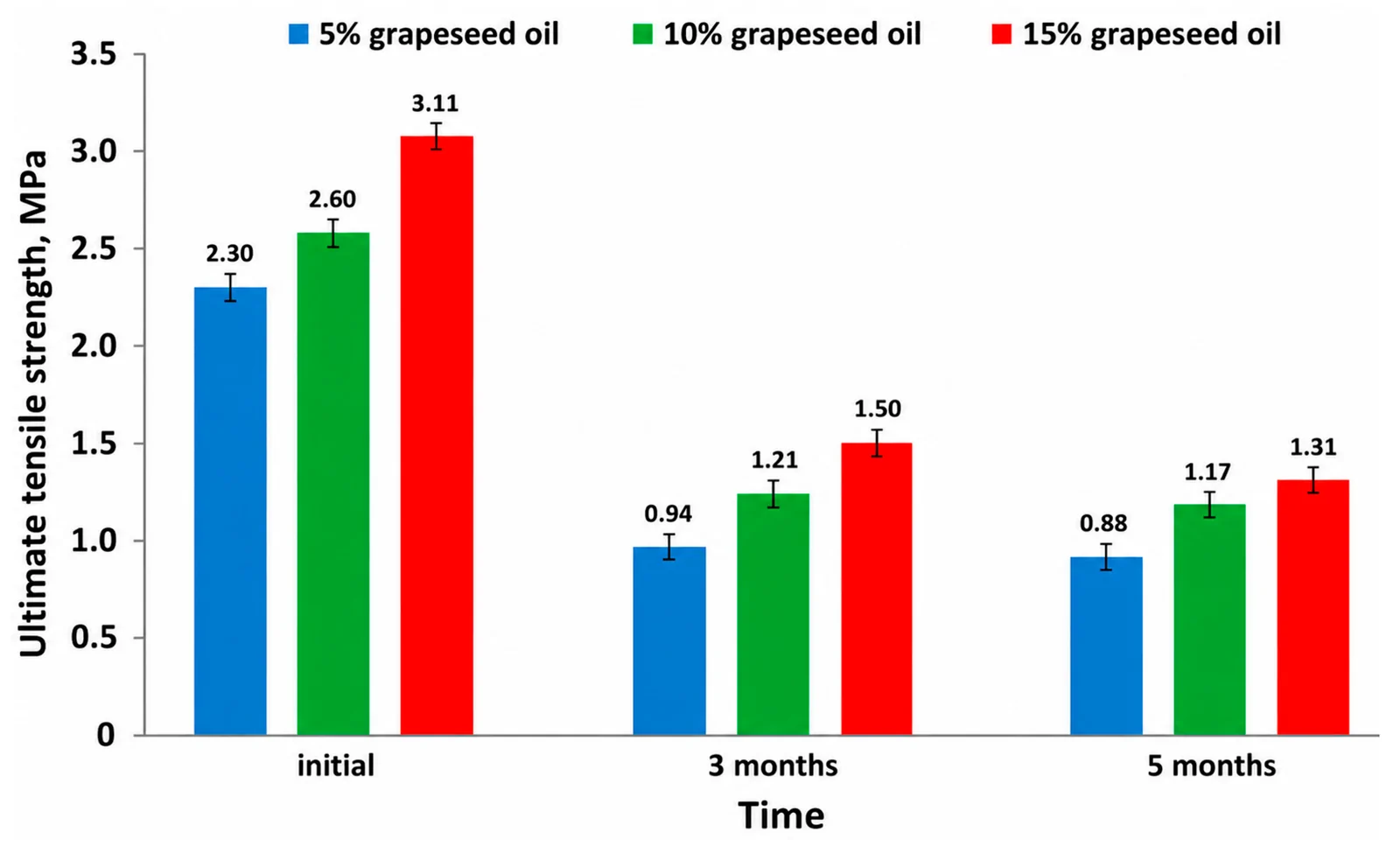
The variation in UTS for the analyzed samples.

**Figure 11:**
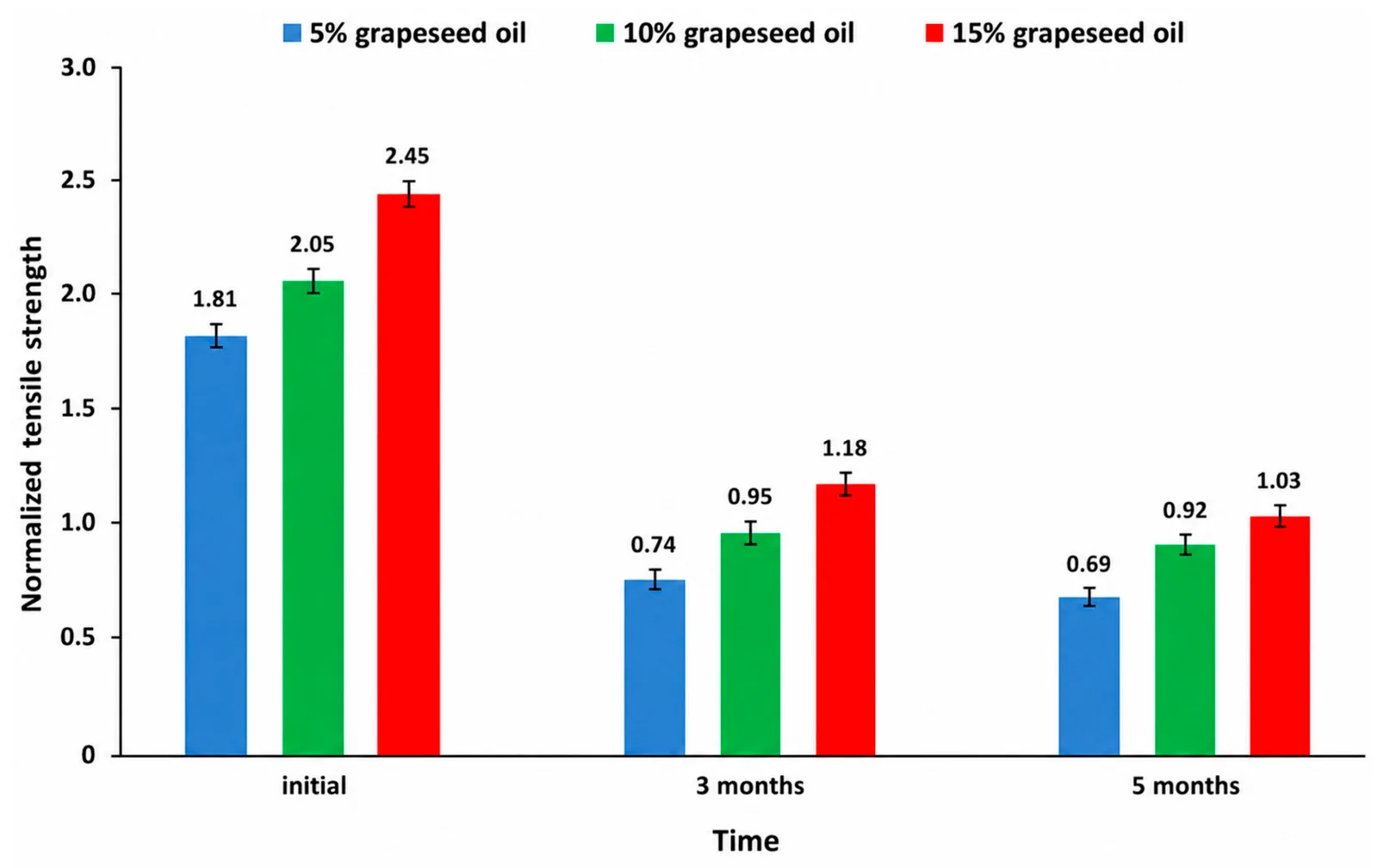
Normalized tensile strength.

The tensile testing results presented in Figure 10 and Figure 11 highlight a clear dependence of the mechanical performance of starch-based films on both grapeseed oil content and storage time. In the initial state, the incorporation of grapeseed oil leads to a pronounced increase in ultimate tensile strength (UTS) compared to the oil-free reference sample. While the neat starch film exhibits a relatively low UTS of 1.27 MPa, the addition of 5%, 10%, and 15% grapeseed oil increases the tensile strength to 2.3 MPa, 2.6 MPa, and 3.11 MPa, respectively. This corresponds to normalized stress values up to 2.44 for the 15% oil formulation, indicating a more than twofold improvement in tensile resistance.

This strengthening effect can be attributed to improved intermolecular interactions within the starch matrix at moderate oil contents. Similar trends have been reported in the literature for starch films plasticized or modified with vegetable oils, where lipid components enhance film cohesion by promoting better stress transfer and reducing microstructural discontinuities. Studies on starch films [29,90,91] commonly report UTS values in the range of 1–4 MPa, placing the present results well within the typical mechanical performance of starch-based materials.

In contrast to Mentha pulegium [92] modified starch films, where increasing the essential oil content from 1% to 2% (*v*/*v*) caused a statistically significant decrease in tensile strength (*p* < 0.05), followed by no further significant change between 2% and 3% (*v*/*v*), the present study shows a clear initial strengthening effect with increasing grapeseed oil content. A strengthening effect similar in direction has been reported for cinnamon essential oil (CEO)-modified polyvinyl alcohol (PVA) and pinto bean starch (PBS) films, where the addition of 1–3% CEO significantly increased tensile strength from 43.2 MPa to 52.0 MPa, attributed to plasticization and improved polymer chain mobility.

These contrasting behaviors confirm that the effect of lipid addition on film mechanical performance depends on both the characteristics of the lipid and its ability to interact with the polymer matrix [93]. However, the beneficial effect of grapeseed oil diminishes with storage time. After three months, all oil-containing samples exhibit a substantial reduction in UTS, with normalized stress values decreasing to 0.74–1.18 depending on oil content. After five months, the tensile strength stabilizes at even lower levels, particularly for the 5% oil sample, which drops below the strength of the oil-free reference. This mechanical degradation over time is consistent with aging phenomena reported for starch-based films; for instance, in study [94] it was reported that after two weeks of storage, the tensile strength decreased to less than 50% of the values measured in the absence of sorbate.

Notably, higher oil contents (10% and 15%) retain tensile strengths close to or slightly above the reference sample even after five months. This indicates that, although aging negatively affects all formulations, a higher grapeseed oil fraction may partially eliminate long-term mechanical deterioration by maintaining a more flexible and energy-dissipating microstructure.

**Figure 12:**
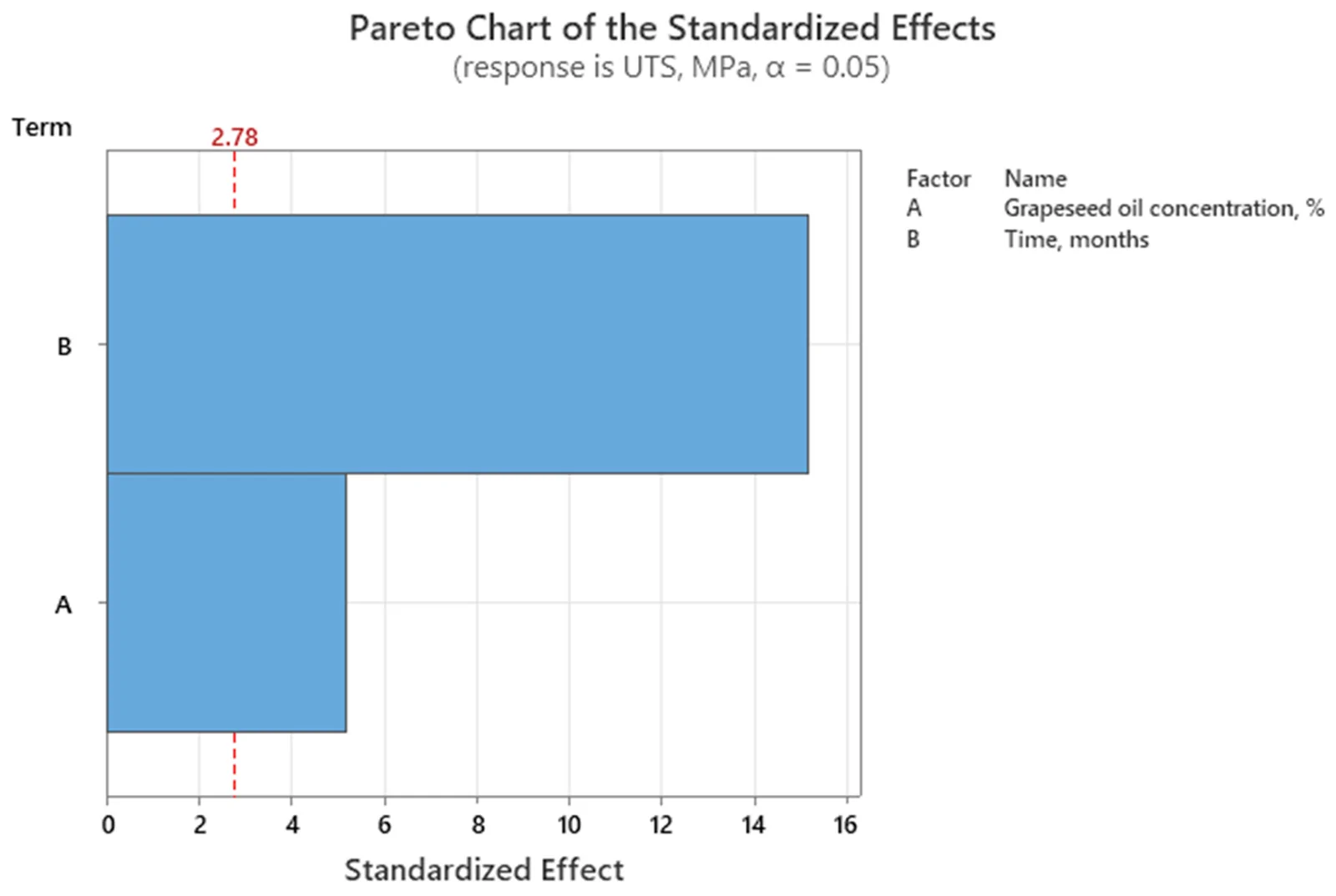
Pareto chart.

The Pareto chart of standardized effects (Figure 12) indicates that storage time (Factor B) is the dominant parameter influencing the ultimate tensile strength (UTS) of the starch–grapeseed oil films. The standardized effect of time is well above the significance threshold (α = 0.05, critical value ≈ 2.78), demonstrating a statistically significant and strong negative impact on UTS. This confirms that aging plays a decisive role in the mechanical degradation of the films.

In contrast, grapeseed oil concentration (Factor A) shows a much smaller standardized effect, remaining close to or slightly below the significance limit. This indicates that, although oil content affects tensile strength—particularly in the initial state—its influence is considerably weaker than that of storage time when both factors are considered simultaneously.

**Figure 13:**
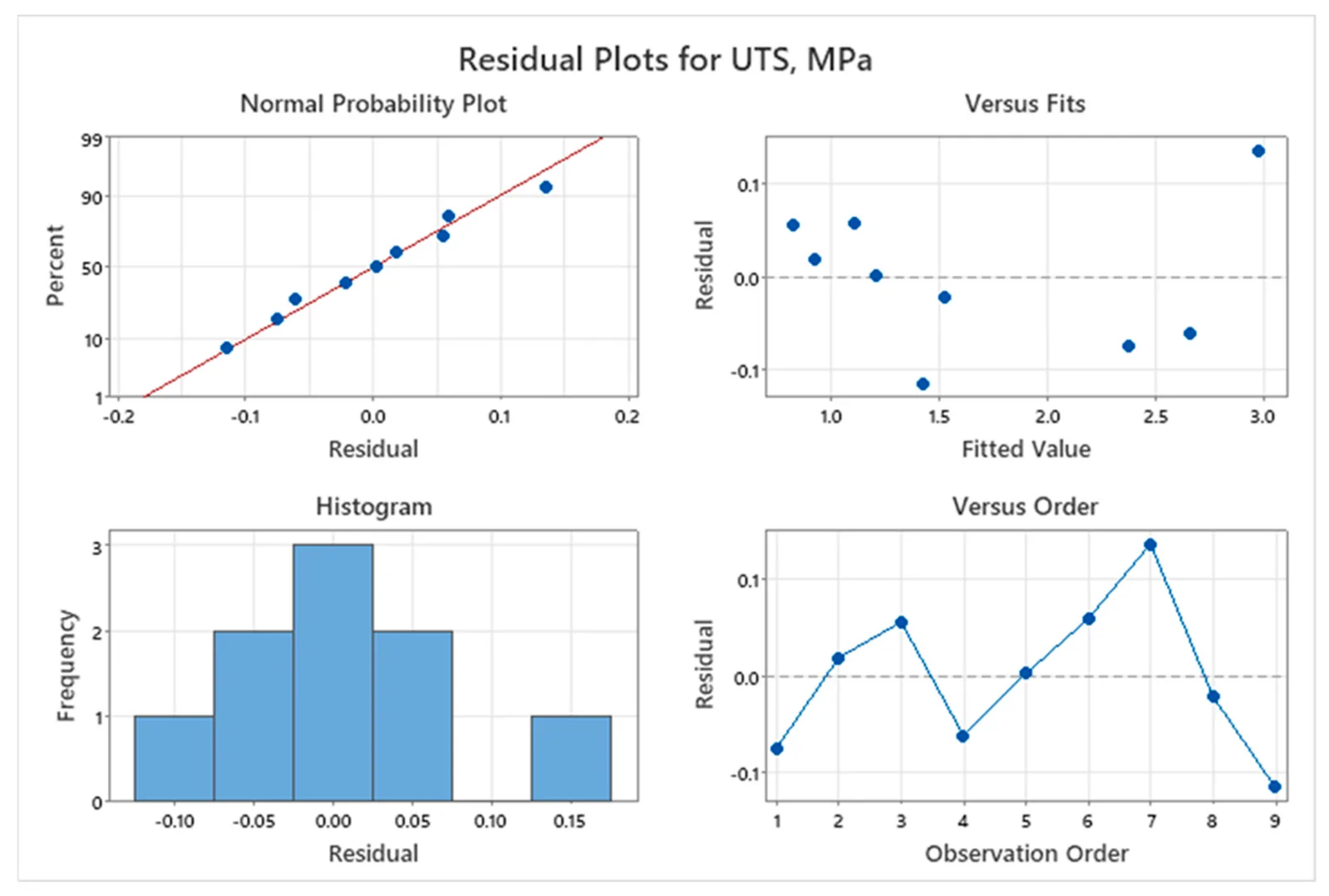
Residual plots.

The residual plots from Figure 13 indicate that the statistical model provides an adequate fit to the experimental data. The normal probability plot shows that the residuals closely follow a straight line, indicating that they are approximately normally distributed, with no evident outliers. This observation is further supported by the histogram, which is centered around zero and displays a reasonably symmetric distribution. The residuals versus fitted values plot does not reveal any systematic pattern or funnel-shaped trend, indicating homoscedasticity and confirming that the variance in the residuals remains constant across the range of predicted UTS values. Similarly, the residuals versus the observation order plot shows random dispersion around zero, with no apparent trends, confirming the absence of autocorrelation.

The analysis of variance (Table 5) confirms that the proposed linear model is highly significant (*p* < 0.001), explaining 99.06% of the total variation in ultimate tensile strength (UTS). Both investigated factors—grapeseed oil concentration and storage time—exert a statistically significant influence on UTS. However, their relative contributions differ markedly. Storage time is the dominant factor, accounting for 88.49% of the total variance, with a very high F-value of 188.07 (*p* < 0.001), indicating a strong time-dependent degradation of tensile strength. In comparison, grapeseed oil concentration contributes 10.57% to the overall variability and remains statistically significant (*p* = 0.007), confirming its secondary but non-negligible role in determining mechanical performance.

The low error contribution (0.94%) and the small mean square error further indicate good experimental consistency and limited unexplained variability. The model summary in Table 6 supports these findings, showing excellent goodness-of-fit metrics, with an R^2^ of 99.06% and an adjusted R^2^ of 98.12%. The predicted R^2^ value of 95.24% is in close agreement with the adjusted R^2^, demonstrating strong predictive capability and minimal overfitting.

## 5. Statistical Analysis of the Experimental Data

**Figure 14:**
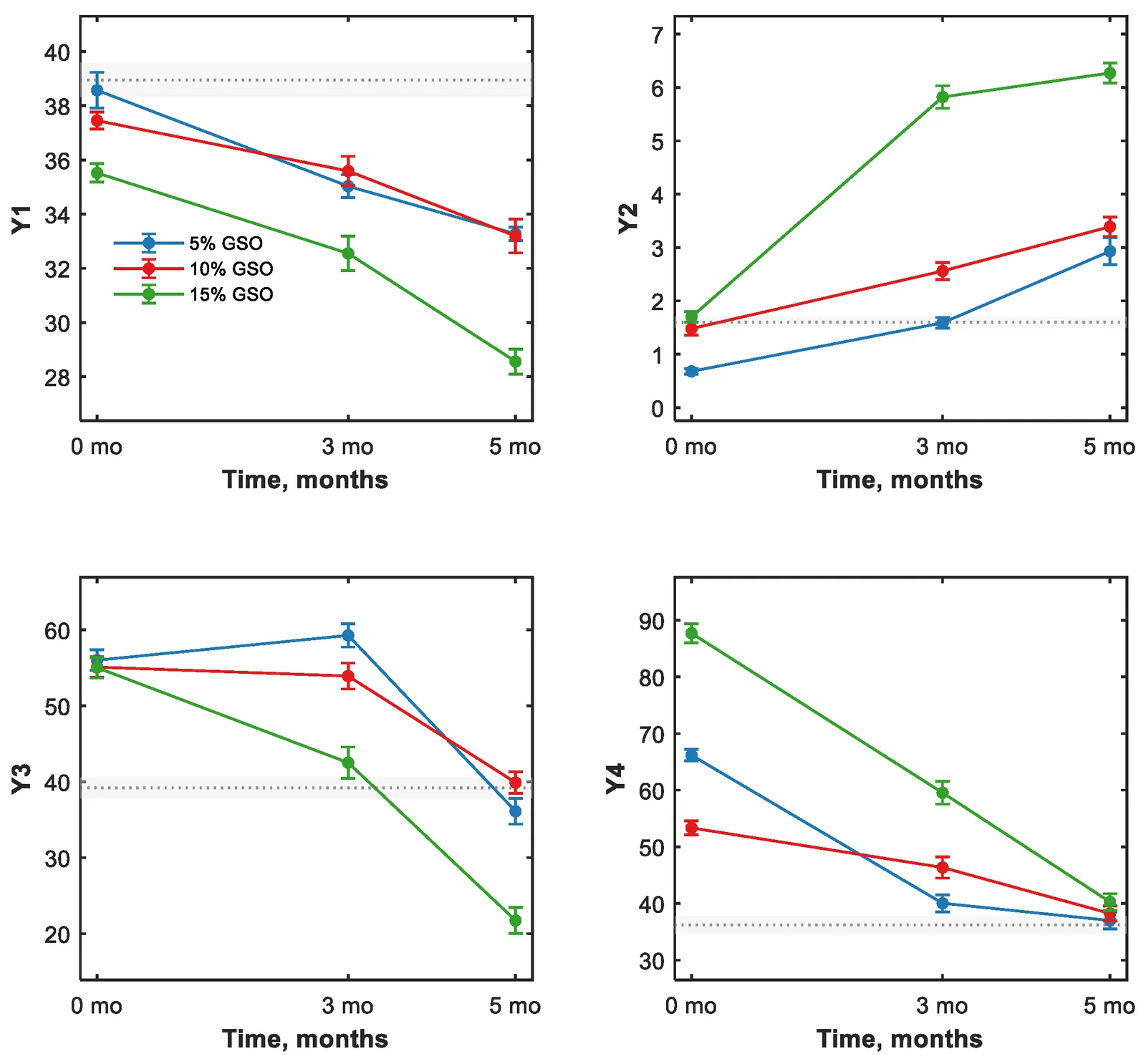
Experimental responses and their corresponding error bars.

To analyze the acquired experimental results on a statistical basis, the responses were denoted in the following way: solubility (Y1), opacity (Y2), swelling index (Y3), and contact angle (Y4). Figure 14 shows the effect of grape seed oil (GSO) concentration on responses from Y1 to Y4 over time (0, 3 and 5 months), compared against baseline values, horizontal dotted lines and shaded error bands. Based on these data, one can observe that Y1 decreases over time for all experimental groups. A higher GSO concentration (15%) causes the greatest reduction, keeping Y1 values significantly below the baseline control range. The results for Y2 display a time-dependent increase across all formulations, with 15% GSO showing the most pronounced elevation compared to 5% and 10% GSO and the baseline. Results for Y3 remain relatively stable up to 3 months for 5% and 10% GSO before dropping sharply at 5 months. The 15% GSO group exhibits a continuous decline throughout the study period, falling below the control baseline by month 3. Given these results, 15% GSO induces the greatest change over time across all measured parameters compared to 5% and 10%.

### 5.1. Two-Way ANOVA Analysis

To evaluate the effects of formulation concentration and storage duration on the evaluated parameters (Y1 to Y4), a two-way analysis of variance was carried out using the experimentally available data. The experimentally available data (nine experiments) were baseline-normalized relative to the untreated control (0 mo, 0% GSO). A fixed-effect two-way ANOVA was carried out on the baseline-shifted response (ΔY) for each outcome variable. The linear statistical model was parametrized as follows [95]:

$$\Delta Y_{ijk}=\mu +\alpha_{i}+\beta_{j}+{\left(\alpha \beta \right)}_{ij}+\epsilon_{ijk}$$

where μ is the overall mean, $\alpha_{i}$ is the main effect of time, $\beta_{j}$ is the main effect of GSO concentration, ${\left(\alpha \beta \right)}_{ij}$ is the two-factor interaction effect, and $\epsilon_{ijk}\sim N\left(0,\sigma^{2}\right)$ is the random experimental error. The normality of the standardized model residuals across all 27 observations was evaluated using normal probability plots (see Figure S1). This plot confirms that the assumption of normality is reasonably met to justify the parametric two-way ANOVA tests for all responses. The results for two-way ANOVA analyses are provided in Tables S1–S4. These analyses show that time, concentration and their binary interaction significantly influence all responses from Y1 to Y4. The interaction between these two factors shows that the direction of change over time depends on the specific GSO concentration applied. All calculations were carried out using the MATLAB 2026a programming environment.

### 5.2. Multivariate Analysis of Variance (MANOVA)

**Figure 15:**
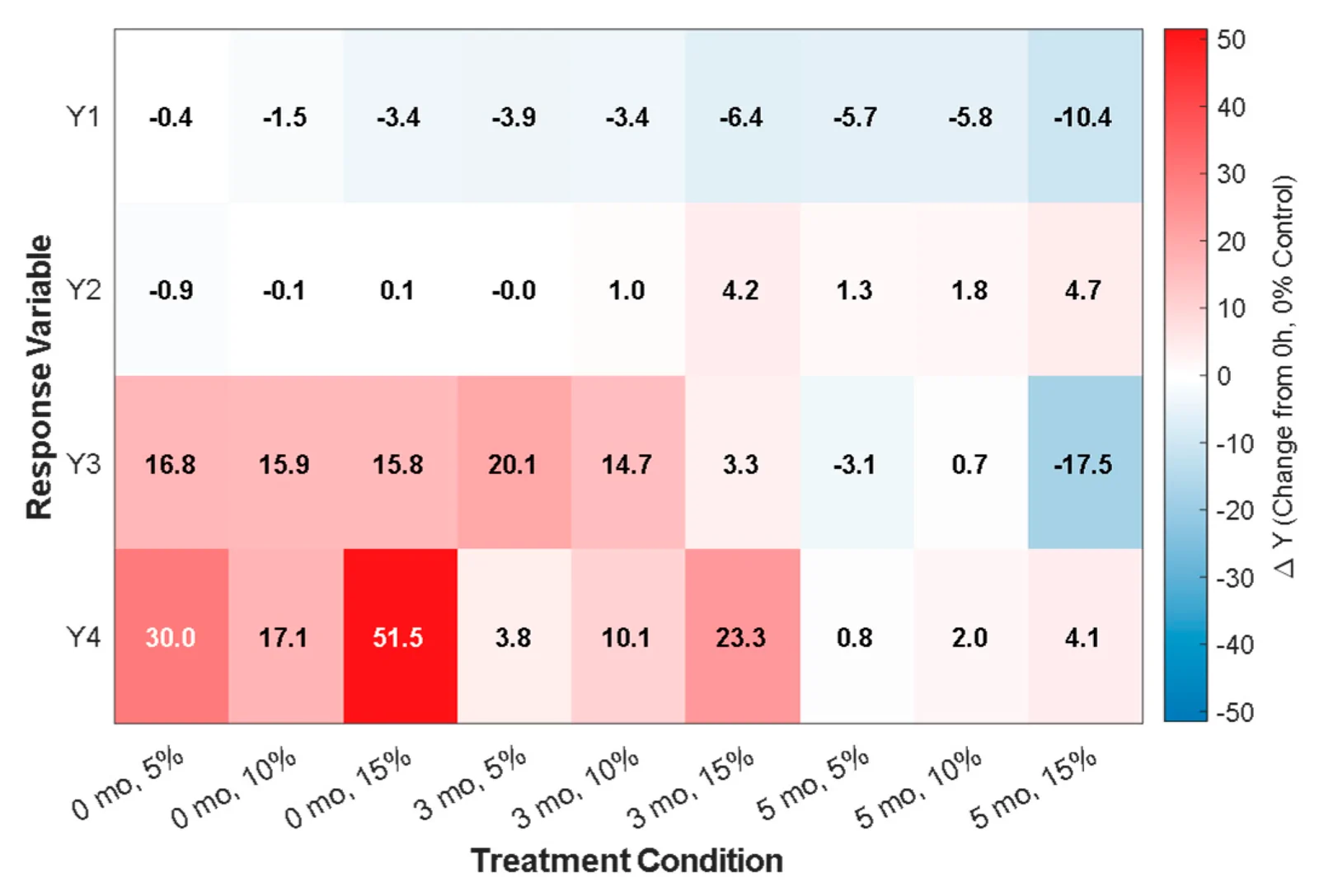
MANOVA-generated heatmap.

To assess the simultaneous effect of duration and GSO concentration across all measured responses, a one-way multivariate analysis of variance approach was carried out. The response matrix was built using the baseline-normalized variables (as outlined in the previous paragraph). Individual experimental groups were defined by combinations of time and concentration levels (i.e., 3 mo, 10%). Canonical discriminant analysis was performed to determine the dimensionality of the space, including the group means, using a significance level of α=0.05 and hypothesis testing for equality of multivariate group means based on the *p*-value criterion was carried out using the MATLAB 2026a programming environment [96]. The results of this analysis are reported in Table S5, Figure 14 and Figure S2. Given that all the tested dimensions in Table S5 are significant, the multi-response changes caused by GSO concentration and time are complex and distributed across multiple independent patterns. Based on both the heatmap (Figure 15) and grouped bar charts (Figure S2), one can draw several conclusions: response variable Y1 shows a consistent negative shift (ΔY) as time increases. The reduction is the most severe in the fifth month under the highest concentration, with the minimum value (−10.4) reached at a time of 5 months and a GSO concentration of 15%.

Response Y4 shows a positive shift at the initial application but at prolonged storage times (3 and 5 months); high values are reduced across all concentration levels. Response variable Y3 maintains positive elevation values through early and intermediate determinations, with the highest value of +20.1 at 3 months’ time and a GSO concentration of 5%. However, at 5 months’ time and the highest concentration (GSO 15%), Y3 shows a sharp inversion into a negative shift (ΔY= −17.5) indicating a possible chemical modification of the sample. Response variable Y2 remains relatively stable near baseline levels during early time points. Relatively small positive increases are shown at later stages, reaching ΔY= 4.7 at 5 months’ time and a GSO concentration of 15%.

## 6. Conclusions

Grape seed oil significantly influenced the initial performance and long-term behavior of potato-starch-based films. Increasing the GSO concentration up to 15% improved the initial tensile strength from 1.27 MPa for the oil-free film to 3.11 MPa, while the maximum degradation temperature reached 319.33 °C and the oxidation onset temperature increased from 60.54 °C to 93.81 °C, demonstrating improved thermal and thermo-oxidative stability. The most important finding, however, is that storage time was the dominant factor governing the long-term mechanical performance of the films, accounting for 88.49% of the variation in tensile strength, compared with 10.57% for GSO concentration. Although aging induced changes in the mechanical, thermal, and structural characteristics of the films, formulations containing higher GSO concentrations (10–15%) showed better retention of mechanical performance after prolonged storage. These findings indicate that GSO can be considered a promising functional component for starch-based biodegradable packaging, although formulation optimization remains necessary to ensure long-term stability. Future studies should focus on the influence of controlled temperature and relative humidity, barrier and migration properties, biodegradation behavior, and validation of the optimized films in real food-packaging systems.

## Figures and Tables

**Table 1:** Sample labeling and grape seed oil composition.

Sample	Storage Time	Grape Seed Oil (%)
SF-0-0	0 months	0
SF-0-5	0 months	5
SF-0-10	0 months	10
SF-0-15	0 months	15
SF -3-5	3 months	5
SF-3-10	3 months	10
SF-3-15	3 months	15
SF-5-5	5 months	5
SF-5-10	5 months	10
SF-5-15	5 months	15

**Table 2:** Thermal stability of starch films.

Sample	Storage Time	Stage 1 (25–120 °C)	Stage 2 (120–360 °C)	Residual Mass (%)	T_max_ (°C)	Onset Temperature (°C)
SF-0-0	0 months	9.86	74.01	16.13	294	269.91
SF-5-5	5 months	4.52	77.27	19.87	301	280.60
SF-5-10	5 months	4.07	79.21	18.65	304	288.04
SF-5-15	5 months	4.4	74.55	21.05	309.33	305.60
SF-3-5	3 months	3.97	77.27	18.76	309.17	296.51
SF-3-10	3 months	3.11	77.36	19.53	314.33	300.01
SF-3-15	3 months	2.08	72.94	24.98	311.83	293.17
SF-0-5	0 months	2.86	73.48	22	310	290.94
SF-0-10	0 months	2.14	75.14	20.79	318.67	300.99
SF-0-15	0 months	1.59	66.37	32.04	319.33	303.34

**Table 3:** DSC data of the starch films.

Sample	Storage Time	T_m_ (°C)	OOT (°C)
SF-0-0	0 months	68.57	60.54
SF-5-5	5 months	78.27	66.37
SF-5-10	5 months	83.57	70.21
SF-5-15	5 months	89.13	87.07
SF-3-5	3 months	84.72	68.22
SF-3-10	3 months	100	76.21
SF-3-15	3 months	93.59	91.57
SF-0-5	0 months	104.5	68.73
SF-0-10	0 months	117.63	77.00
SF-0-15	0 months	118.49	93.81

**Table 4:** Lattice parameters and degree of crystallinity of starch β-type (potato starch) (space group P61).

Sample	Storage Time	Xc, XRD (%)	a = b, nm	c, nm	Miller Indices
SF-0-0	0 months	22.87	1.83	1.01	
SF-5-5	5 months	9.21	1.86	1.04	(010), (111), (212), (140), (013), (240), (233), (333), (063), (443), (632)
SF-5-10	5 months	11.25	1.83	1.05	(010), (240)
SF-5-15	5 months	10.07	1.85	1.03	(010), (212), (240)
SF-3-5	3 months	11.03	1.79	1.01	(010), (212), (240), (063)
SF-3-10	3 months	10.49	1.87	1.05	(010), (212), (013), (240)
SF-3-15	3 months	11.82	1.84	1.07	(010)
SF-0-5	0 months	11.11	1.80	1.06	(010), (240)
SF-0-10	0 months	11.76	1.84	1.03	(010)
SF-0-15	0 months	12.48	1,82	1.04	(010), (240)

**Table 5:** Analysis of variance parameters.

Source	DF	Seq SS	Contribution	Adj SS	Adj MS	F-Value
Model	4	5.06438	99.06%	5.06438	1.26609	105.26
Linear	4	5.06438	99.06%	5.06438	1.26609	105.26
Grapeseed oil concentration, %	2	0.54036	10.57%	0.54036	0.27018	22.46
Time, months	2	4.52402	88.49%	4.52402	2.26201	188.07
Error	4	0.04811	0.94%	0.04811	0.01203	
Total	8	5.11249	100.00%			

**Table 6:** Model summary.

S	R-sq	R-sq (Adj)	PRESS	R-sq (Pred)
0.109671	99.06%	98.12%	0.243562	95.24%

## Data Availability

Data will be made available on request.

## Supplementary Materials

The following supporting information can be downloaded at https://www.mdpi.com/article/10.3390/polym18182279/s1.
