# Supplementary material for: Evaluation of the Storage Stability of Starch-Based Films Containing Grape Seed Oil
Source: Polymers (Basel). 2026 Sep 17;18(18):2279. doi: 10.3390/polym18182279 (PMC13611048; doi:10.3390/polym18182279)
Supplement: Supplementary file 1 [file polymers-18-02279-s001.zip › polymers-4503313-supplementary.pdf]

Supplementary Material

Determination of Film Swelling Degree

Film swelling was determined by weighing the dried film samples (MDry) and immersing them individually in distilled water for 24 h at room temperature. After immersion, the samples were removed, gently blotted with filter paper to eliminate excess surface water, and weighed again. The swelling degree was calculated according to:

$$\text{Swelling degree (\%)} = (\text{MSwollen} - \text{MDry})/\text{MDry} \times 100 \qquad \text{(S1)}$$

where MDry = the initial mass of the polymer sample, and MSwollen = mass of the sample after swelling.

Determination of Film Solubility in Water

Film solubility was evaluated by weighing the dried samples (Wi), immersing them in distilled water for 24 h, and subsequently drying the undissolved residues in an oven at 105 °C for 48 h. The final dry mass (Wf) was recorded, and the water solubility was calculated as:

$$\text{Solubility (\%)} = [W_i - W_f] \times 100/W_i \qquad \text{(S2)}$$

Water Contact Angle

The water contact angle was measured using the sessile drop method. A distilled water droplet was carefully deposited onto the film surface, and images of the droplet were captured 10 s after deposition. The contact angle was determined using **ImageJ** software (version 1.54).

Statistical analysis data

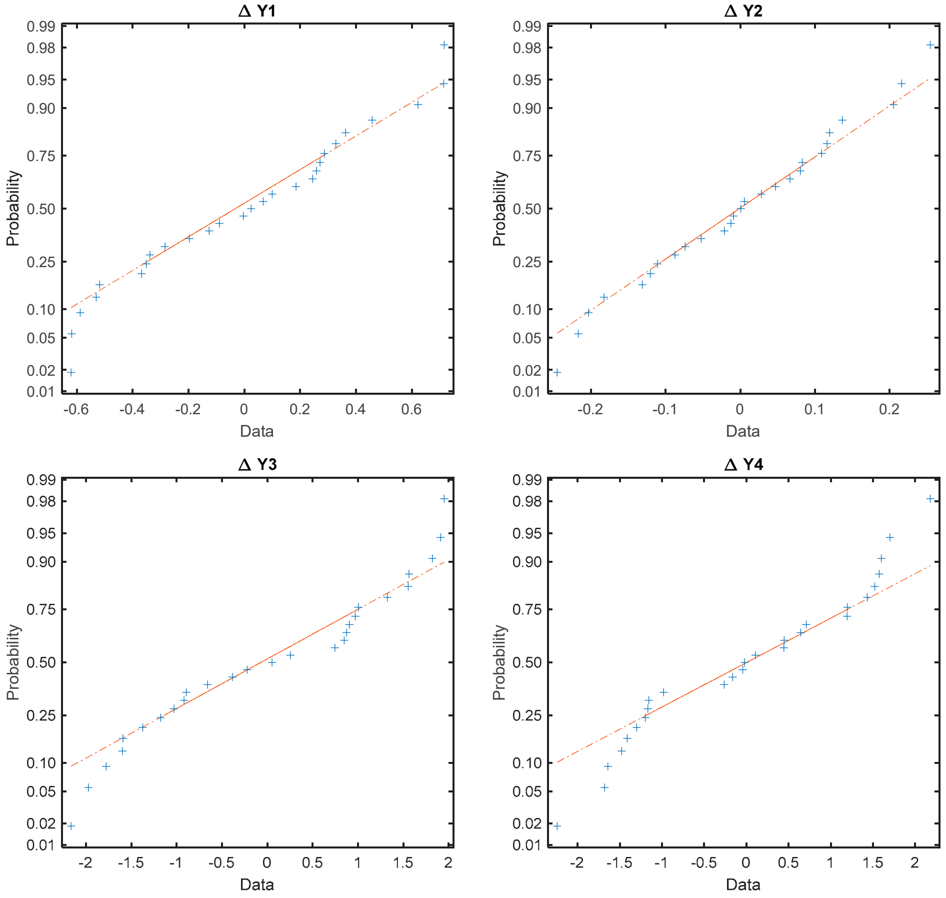

Figure S1. Normal probability plots for baseline-shifted responses

Table S1. Two-Way ANOVA Table for ΔY1

**Source**	**Sum Sq.**	**d.f.**	**Mean Sq.**	**F**	**Prob>F**
Time	136.4	2	68.2	281.2	$2.65\cdot10^{-14}$
Conc	65.8	2	32.9	135.6	$1.40\cdot10^{-11}$
Time:Conc	7.8	4	1.9	8.1	$7.00\cdot10^{-4}$
Error	4.3	18	0.2		
Total	214.4	26			

Table S2. Two-Way ANOVA Table for ΔY2

Source	Sum Sq.	d.f.	Mean Sq.	F	Prob>F
Time	40.14	2	20.07	760.16	$4.11 \cdot 10^{-18}$
Conc	39.74	2	19.87	752.59	$4.49 \cdot 10^{-18}$
Time:Conc	11.11	4	2.78	105.26	$3.15 \cdot 10^{-12}$
Error	0.48	18	0.03		
Total	91.46	26			

Table S3. Two-Way ANOVA Table for ΔY3

Source	Sum Sq.	d.f.	Mean Sq.	F	Prob>F
Time	2716.80	2	1358.40	535.03	$9.28 \cdot 10^{-17}$
Conc	639.02	2	319.51	125.85	$2.63 \cdot 10^{-11}$
Time:Conc	353.78	4	88.45	34.84	$3.01 \cdot 10^{-8}$
Error	45.70	18	2.54		
Total	3755.30	26			

Table S4. Two-Way ANOVA Table for ΔY4

Source	Sum Sq.	d.f.	Mean Sq.	F	Prob>F
Time	4363.1683	2	2181.5	916.8	$7.75 \cdot 10^{-19}$
Conc	1482.8483	2	741.4	311.6	$1.08 \cdot 10^{-14}$
Time:Conc	936.3047	4	234.1	98.3	$5.63 \cdot 10^{-12}$
Error	42.8282	18	2.3		
Total	6825.1495	26			

Table S5. MANOVA analysis results

Tested Dimension	Wilks' Lambda criterion	Approx Chi-sq	df	Prob>ChiSq
$Dimension \geq 0$	$1.76 \cdot 10^{-7}$	303.31	32	$6.01 \cdot 10^{-46}$
$Dimension \geq 1$	$9.31 \cdot 10^{-5}$	181.01	21	$1.89 \cdot 10^{-27}$
$Dimension \geq 2$	$1.08 \cdot 10^{-2}$	88.30	12	$1.05 \cdot 10^{-13}$
$Dimension \geq 3$	0.23	28.39	5	$3.05 \cdot 10^{-5}$

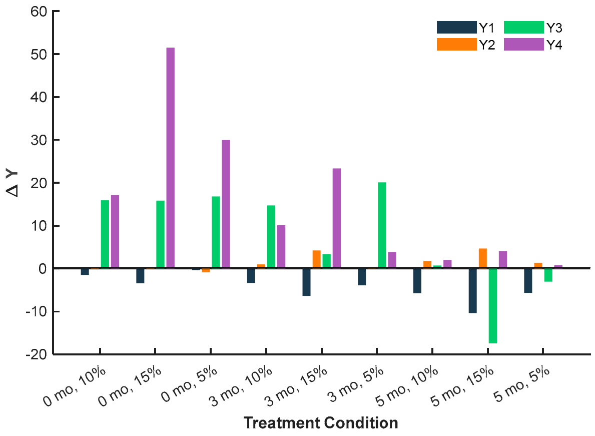

Figure S2. MANOVA generated grouped bar chart
